# Supplementary material for: Is latent tuberculosis infection challenging in Iranian health care workers? A systematic review and meta-analysis
Source: PLoS One. 2019 Oct 3;14(10):e0223335. doi: 10.1371/journal.pone.0223335 (PMC6776393; doi:10.1371/journal.pone.0223335)
Supplement: S2 File — (PDF) [file pone.0223335.s002.pdf]

Prevalence and risk factors of latent tuberculosis infection among health care workers in ?Iran: a systematic review and meta-analysis

Mohammad Hossein YektaKooshali, Farahnaz Movahedzadeh, Alireza Jafari

Citation

Mohammad Hossein YektaKooshali, Farahnaz Movahedzadeh, Alireza Jafari. Prevalence and risk factors of latent tuberculosis infection among health care workers in ?Iran: a systematic review and meta-analysis. PROSPERO 2018 CRD42018117682 Available from:
http://www.crd.york.ac.uk/PROSPERO/display_record.php?ID=CRD42018117682

Review question

What is the overall prevalence of latent tuberculosis infection (LTBI) among health care ?workers (HCWs) in Iran??

What is the prevalence of LTBI among HCWs based on province in Iran??

What is the prevalence of LTBI among HCW based on geographical regions in Iran??

What is the prevalence of LTBI among HCWs based on gender in Iran??

What is the prevalence of LTBI among HCWs based on age in Iran??

What is the prevalence of LTBI among HCWs based on job and work experience in Iran??

What is the prevalence of LTBI among HCWs based on duration of employment in Iran??

What is the prevalence of LTBI among HCWs based on vaccination history in Iran??

What is the prevalence of LTBI among HCWs based on workplaces in Iran??

What is the prevalence of LTBI among HCWs according to laboratory tests (Interferon-gamma (IFN-?) release ?assay (IGRA), and tuberculin skin tests (TST)) in Iran??

What is the prevalence of LTBI among HCWs based on single-step or two-step TST in Iran? ?

What is the prevalence of LTBI among HCWs based on history of TB disease, hospitalization in Iran, and exposure with TB??

What is the prevalence of LTBI among HCWs based on cigarette smoking??

What is the prevalence/frequency of LTBI among HCWs based on? ?sweet diabetes, ?HIV, silicosis, ?renal chronic failure, leukemia, lymphoma, and immunosuppressive drug?

What is the the meta-regression diagram for prevalence and incidence of LTBI among HCWs in Iran based ?on the year??

?How is the the publication bias in the studies of incidence and prevalence??

Searches

The present study will be done based on the meta-analysis of observational studies according with ?epidemiology guidelines, and the PRISMA statement. To maximize sensitivity, a broad search strategy will be ?lead through Persian(national) databases including Scientific Information Database (SID) ??(http://www.sid.ir/), ?Barakat Knowledge Network System (http://health.barakatkns.com), ??(Iranian

Research ?Institute for Information Science and Technology (IranDoc) ??(https://irandoc.ac.ir), Regional ?Information Center for Science and Technology (RICST) ??(http://en.ricest.ac.ir/), Magiran ??(http://www.magiran.com/), Iranian National Library ??(http://www.nlai.ir/)? and international databases: Web of Science, Scopus, PubMed/MEDLINE, Ovid, EMBASE, and the ?Cochrane Library (Cochrane Database of Systematic Reviews) as well as Google ?Scholar search engine for peer-reviewed studies published. The Persian and English language will be used as a filter in national and international databases, respectively. ?The search terms will be adapted for the different databases. Summarizing: ("latent tuberculosis" OR "Latent ?Tuberculoses") AND Iran in title/abstract/keywords. ?Boolean operators (AND & OR) were used to search by a combination of words. All possible standard search terms, medical subject heading (MeSH) terms, ?and MeSH entry terms will be used. The search terms will also be lead without any time limitation till 21st ?November 2018. The authors will then independently analyses the text words ?contained in the title and abstract, and the index terms used to describe the article.

Types of study to be included

Cross-sectional and epidemiological studies

Condition or domain being studied

LTBI is defined as a state of persistent immune response to stimulation by Mycobacterium tuberculosis ?antigens without evidence of clinically manifested active TB. Someone has latent TB if they are infected with ?the TB bacteria but do not have signs of active TB disease and do not feel ill. However, they can develop active ?TB disease in the future. In this study we will evaluate prevalence and risk factors of LTBI among HCWs in ?Iran. ?

Participants/population

This study will concentrate on the population of HCWs with LTBI who are resident in the geographic regions of ?Northern, Southern, Eastern, Western, center, and capital city of Iran. ?

Intervention(s), exposure(s)

The exposure will be the laboratory tests (Interferon-gamma (IFN-?) release assay (IGRA), and tuberculin skin ?tests (TST)) of which confirmed LTBI among HCWs in Iran. ?

Comparator(s)/control

The comparators population will be HCWs who do not have signs of active TB disease and do not feel ill.?

Context

Iran

Main outcome(s)

Prevalence and risk factors of LTBI among HCWs in Iran

Additional outcome(s)

The prevalence of LTBI among HCW in Iran.

The prevalence of LTBI among HCW based on province in Iran.

The prevalence of LTBI among HCW based on geographical regions in Iran.

The prevalence of LTBI among HCW based on gender in Iran.

The prevalence of LTBI among HCW based on age in Iran.

The prevalence of LTBI among HCW based on job and work experience in Iran.

The prevalence of LTBI among HCW based on duration of employment in Iran.

The prevalence of LTBI among HCW based on BCG vaccination history in Iran.

The prevalence of LTBI among HCW based on workplaces in Iran.

The prevalence of LTBI among HCW according to laboratory diagnosis test in Iran.

The prevalence of LTBI among HCW based on single-step or two-step TST in Iran ?.

The prevalence of LTBI among HCW based on history of TB disease, hospitalization in Iran, and exposure with TB.

The prevalence of LTBI among HCWs based on cigarette smoking.

The prevalence/frequency of LTBI among HCWs based on? ?sweet diabetes, HIV, silicosis, renal chronic failure, leukemia, lymphoma and immunosuppressive drug.

The meta-regression diagram for prevalence and incidence of LTBI among HCW in Iran based on the ?year.?

Publication bias in the studies of incidence and prevalence.?

Data extraction (selection and coding)

Enter terms will include author’s name, province, geographical regions, year of published, sample size, ?mean and median age, gender, history of BCG vaccination, history of exposure with TB, history of TB disease, ?laboratory diagnosis tests, job and work experience, duration of employment, workplaces, single-step or two-?step TST, history of hospitalization, cigarette smoking, sweet diabetes, HIV, silicosis, renal chronic failure, ?leukemia, immunosuppressive drug, which will be extracted by two researchers ?independently and blind to the author's name, ?institution, and journal. When required, ?additional information and raw data will be ?asked by contacting the corresponding author or department (by phone call, academic mailing, Fax and etc.).

All stages will be ?evaluated by two independent reviewers. In cases of disagreements among reviewers we will solve that by ?discussion and consensus among the team and expert microbiologist of the research team.?

Risk of bias (quality) assessment

First, we will eliminate of irrelevant studies and next the quality of the final studies will be examined. The ?Newcastle-Ottawa Scale (NOS) checklist will also be used, which divides the studies with a scale of scores of 0 to ??8 from poor to high quality. The studies will also divide into three levels of scoring: ?

?1- Studies with a score of 5 or less: poor quality; ?

?2- Studies with a score of 5-6: medium quality; ?

?3- Studies with a score of 7 to 8: high quality. ?

Articles of medium to high quality will be used in the data analysis.?

Strategy for data synthesis

The current study will be done based on PRISMA statement. The study will also go ahead based on five steps; design ?and search strategy, collection of original and systematic review articles, evaluation of the inclusion and ?exclusion criteria, and finally qualitative evaluation and statistical analysis of data. All steps will be handled by ?two researchers independently and, in the event of any differences in opinion, a specialist will carefully ?investigate the issue.?

We will provide a narrative synthesis of the results from the included studies.?

? ?

The prevalence rate of LTBI among HCWs will be considering as a binomial distribution probability, and the ?variance will be calculated by binomial distribution. To evaluation of heterogeneity, the Cochran test (Q) and I^2 ?index will be used. As we know, I^2 index less than 25% show a low heterogeneity, between 75% -25% show ?average heterogeneity, and more than 75% show considerable heterogeneity.?

Sensitivity analysis will be achieved to research impact of each study based on the total results of the overall ?prevalence, and the assessment of each of the risk factors.?

To evaluate the causes of heterogeneity, subgroup analyses will be performed based on province, single-step or ?two-step TST, laboratory diagnosis tests, job, gender, history of TB disease, history of TB exposure, BCG ?vaccination history and geographical regions. As well, the meta-regression model will be used to determine of ?prevalence rates based on the year of publication. Next, the Begg's test and Egger's test will be done using a ?funnel plot to examine of publication bias. Data analysis will analyze by the Comprehensive Meta-Analysis ??(Version 2). An alpha of 0.05 will be used as the cutoff for significance. If the p-value is less than 0.05, we will ?reject the null hypothesis that there's no difference between the means and conclude that a ?significant difference does exist.?

Analysis of subgroups or subsets

The following subgroup analyses will be done:?

The prevalence of LTBI among HCW based on province in Iran

The prevalence of LTBI among HCW based on geographical regions in Iran

The prevalence of LTBI among HCW based on gender in Iran

The prevalence of LTBI among HCW based on history of TB exposure in Iran

The prevalence of LTBI among HCW based on BCG vaccination history in Iran

The prevalence of LTBI among HCW according to laboratory diagnosis test in Iran

The prevalence of LTBI among HCW based on single-step or two-step TST in Iran ?

Contact details for further information

Mohammad Hossein YektaKooshali
Yektakooshali.mh1995@yahoo.co.uk

Organisational affiliation of the review

Guilan University of Medical Sciences, Rasht, Iran

Review team members and their organisational affiliations

Mr Mohammad Hossein YektaKooshali. Student Research committee, School of nursing, Midwifery and Paramedicine, Guilan University of Medical Sciences, Rasht, Iran
Dr Farahnaz Movahedzadeh. Department of Medicinal Chemistry and Pharmacognosy, Institute for Tuberculosis Research, College of Pharmacy, University of Illinois at Chicago, Chicago, Illinois, USA
Dr Alireza Jafari. Inflammatory Lung Disease Research Center, Department of Internal Medicine, Razi Hospital, School of ?Medicine, Guilan University of Medical Sciences, Rasht, Iran

Collaborators

Dr Aboozar Ramezani. Department of Medical Library and Information, Virtual School, Tehran University of Medical Sciences, ?Tehran, Iran
Dr Saeed Sadat Mansouri?. Inflammatory Lung Disease Research Center, Department of Internal Medicine, Razi Hospital, School of ?Medicine, Guilan University of Medical Sciences, Rasht, Iran

Type and method of review

Epidemiologic, Methodology, Systematic review

Anticipated or actual start date
24 November 2018

Anticipated completion date
18 January 2019

Funding sources/sponsors
None

Conflicts of interest

Language
English

Country
Iran

Stage of review
Review Ongoing

Subject index terms status
Subject indexing assigned by CRD

Subject index terms
Health Personnel; Humans; Iran; Latent Tuberculosis; Prevalence; Risk Factors

Date of registration in PROSPERO
14 December 2018

Date of publication of this version
14 December 2018

Details of any existing review of the same topic by the same authors

Stage of review at time of this submission

Stage	Started	Completed
Preliminary searches	Yes	No
Piloting of the study selection process	Yes	No
Formal screening of search results against eligibility criteria	No	No
Data extraction	No	No
Risk of bias (quality) assessment	No	No
Data analysis	No	No

Versions
14 December 2018

PROSPERO
This information has been provided by the named contact for this review. CRD has accepted this information in good faith and registered the review in PROSPERO. The registrant confirms that the information supplied for this submission is accurate and complete. CRD bears no responsibility or liability for the content of this registration record, any

associated files or external websites.
