# Supplementary figures and images for: Is latent tuberculosis infection challenging in Iranian health care workers? A systematic review and meta-analysis
Source: PLoS One. 2019 Oct 3;14(10):e0223335. doi: 10.1371/journal.pone.0223335 (PMC6776393; doi:10.1371/journal.pone.0223335)

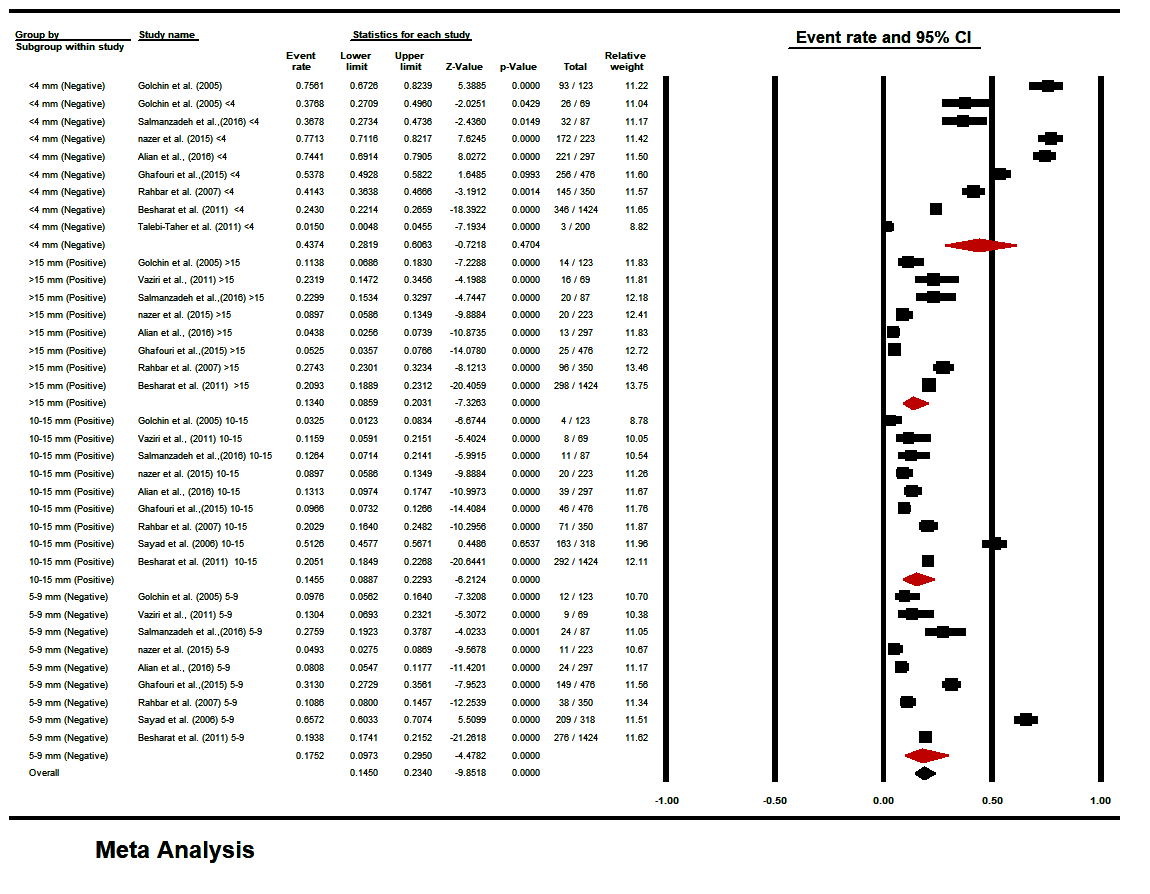

Supplement: S1 Fig — (TIF) [file pone.0223335.s005.tif]

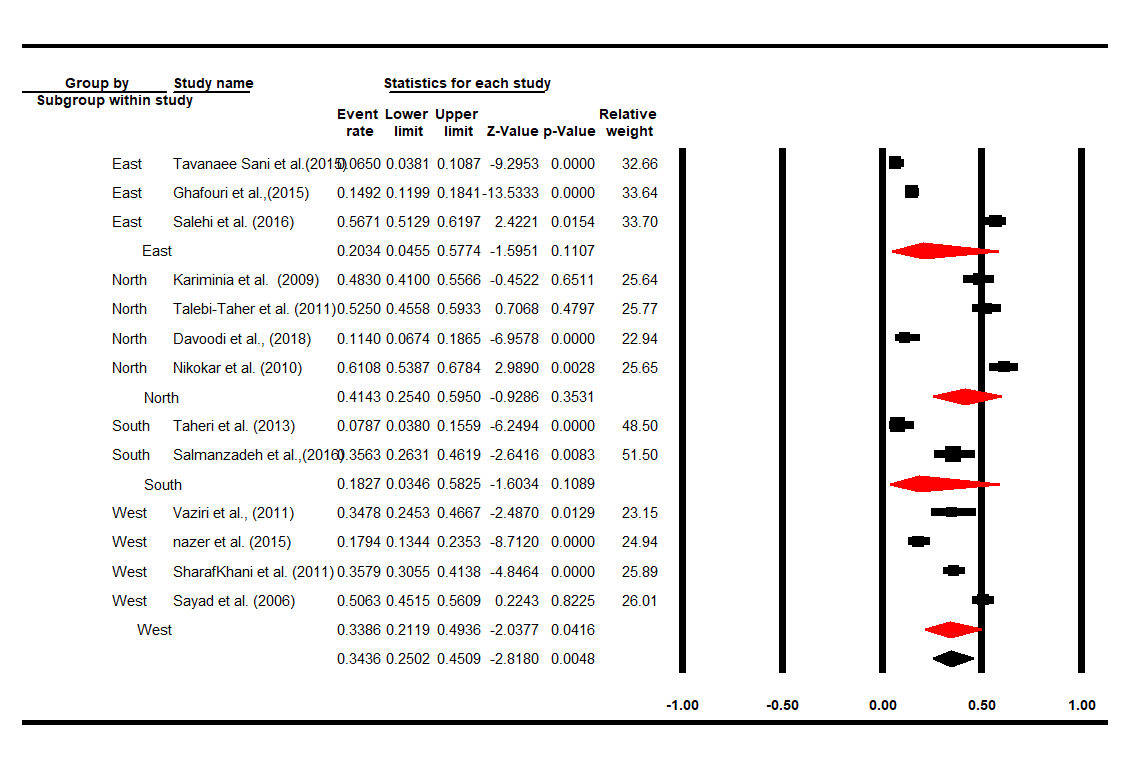

Supplement: S2 Fig — (TIF) [file pone.0223335.s006.tif]

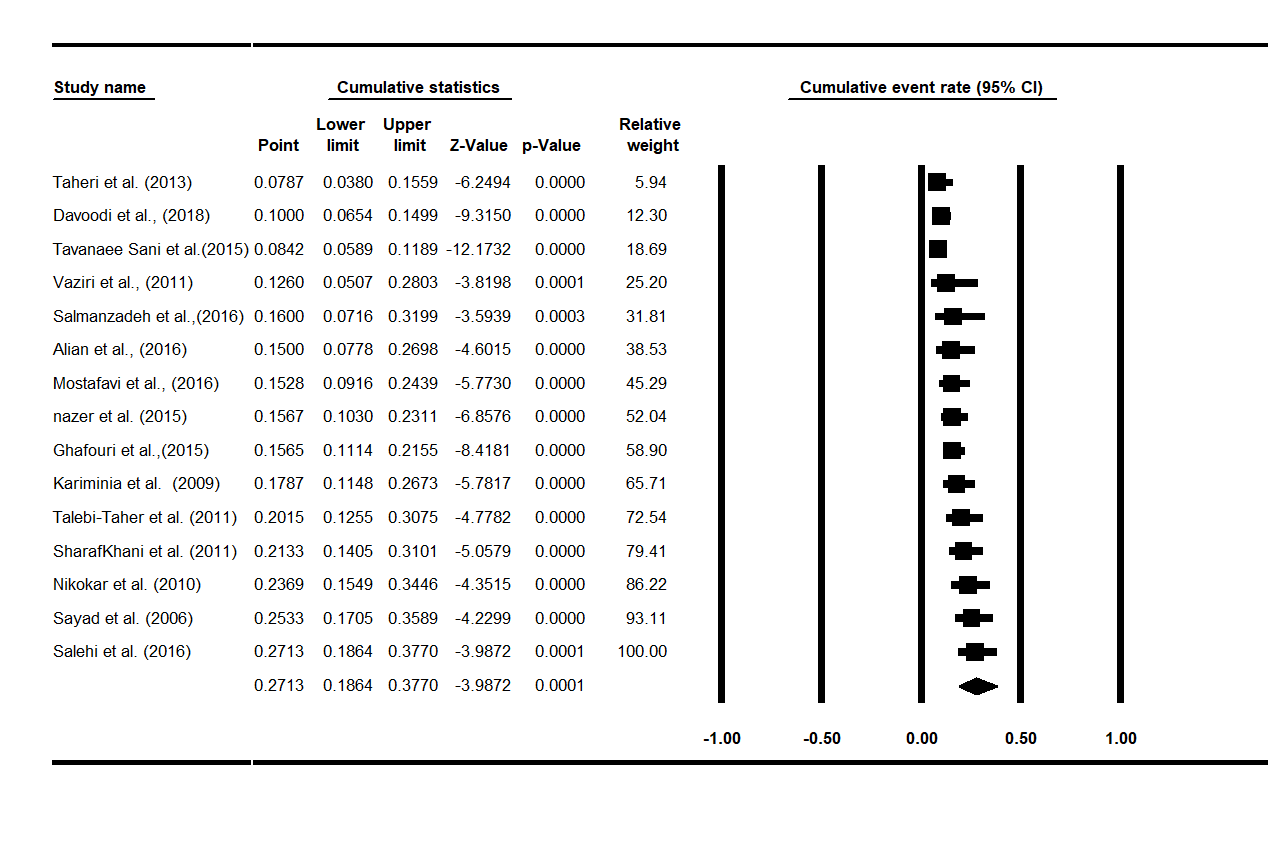

Supplement: S3 Fig — (TIF) [file pone.0223335.s007.tif]

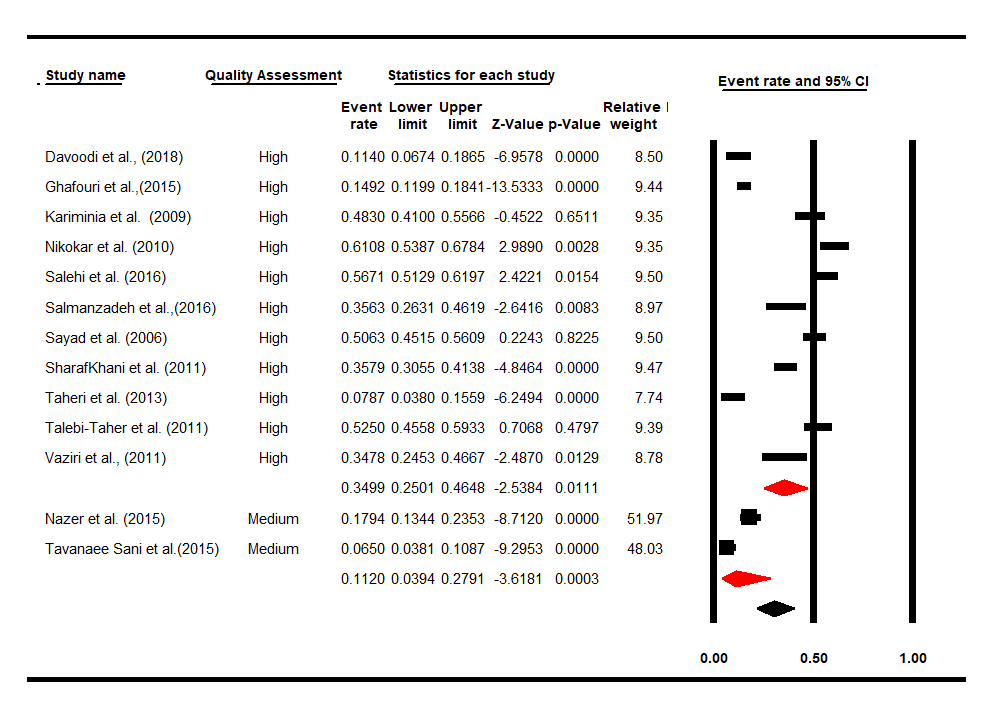

Supplement: S4 Fig — (TIF) [file pone.0223335.s008.tif]

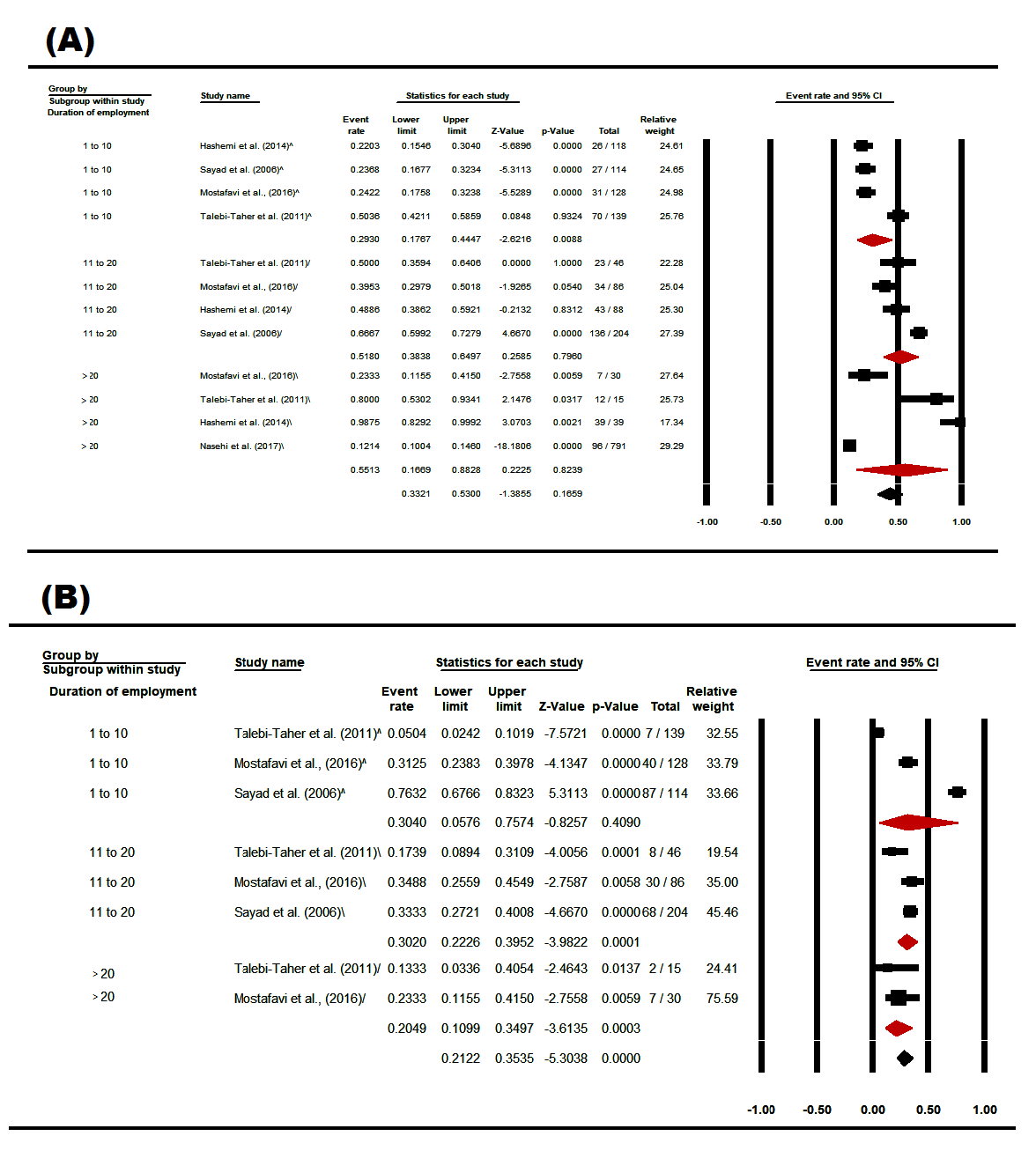

Supplement: S5 Fig — (TIF) [file pone.0223335.s009.tif]

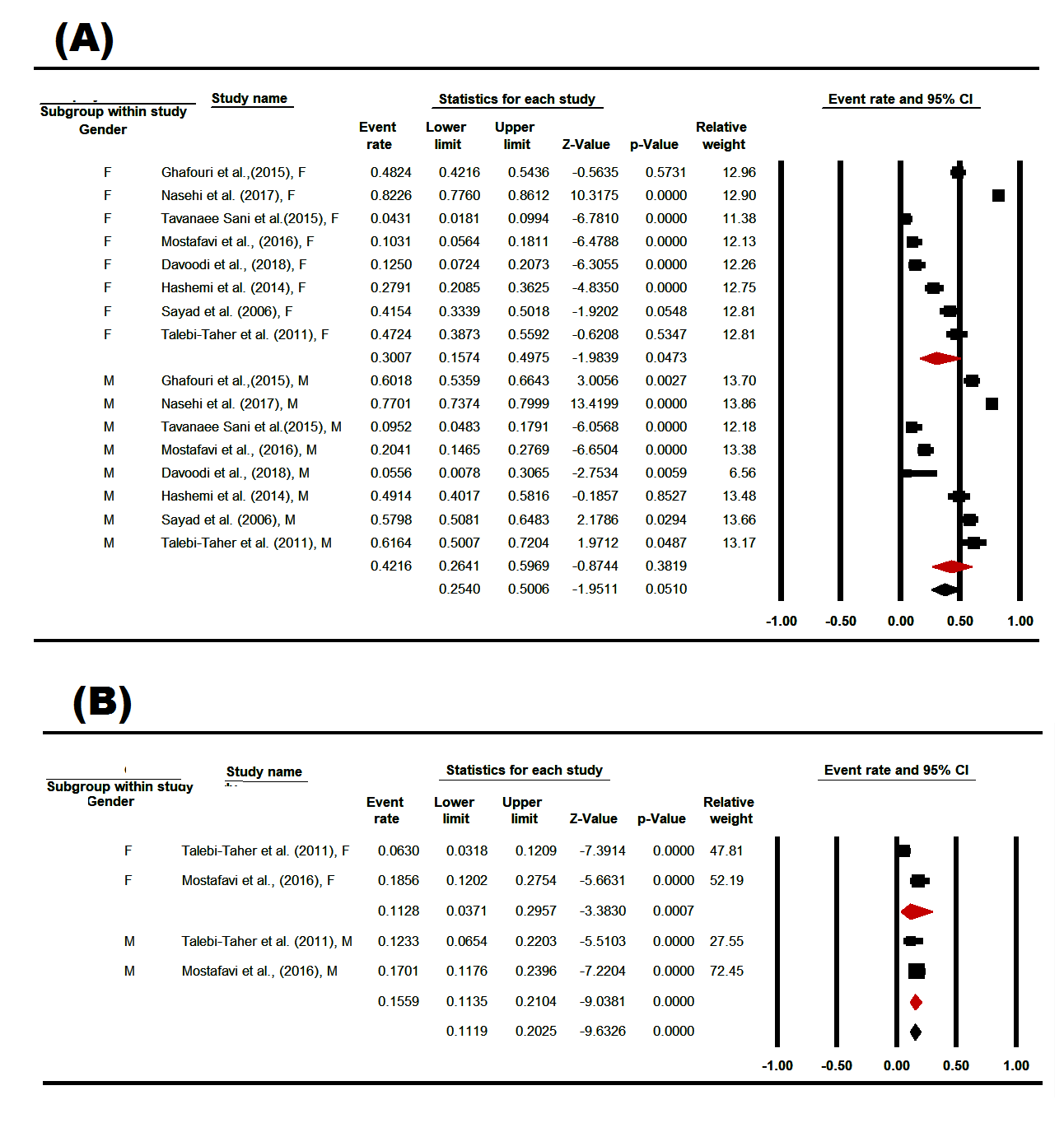

Supplement: S6 Fig — (TIF) [file pone.0223335.s010.tif]

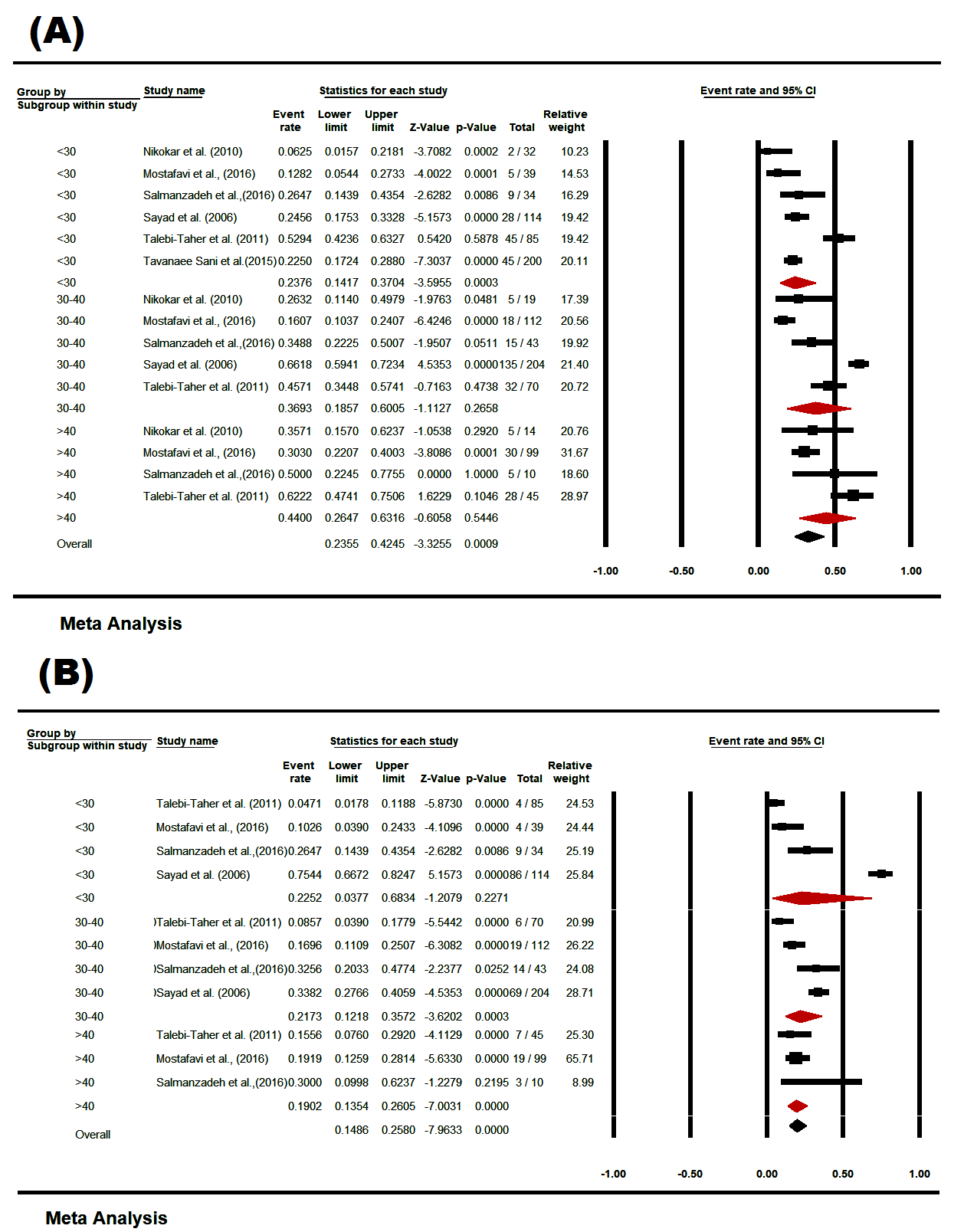

Supplement: S7 Fig — (TIF) [file pone.0223335.s011.tif]

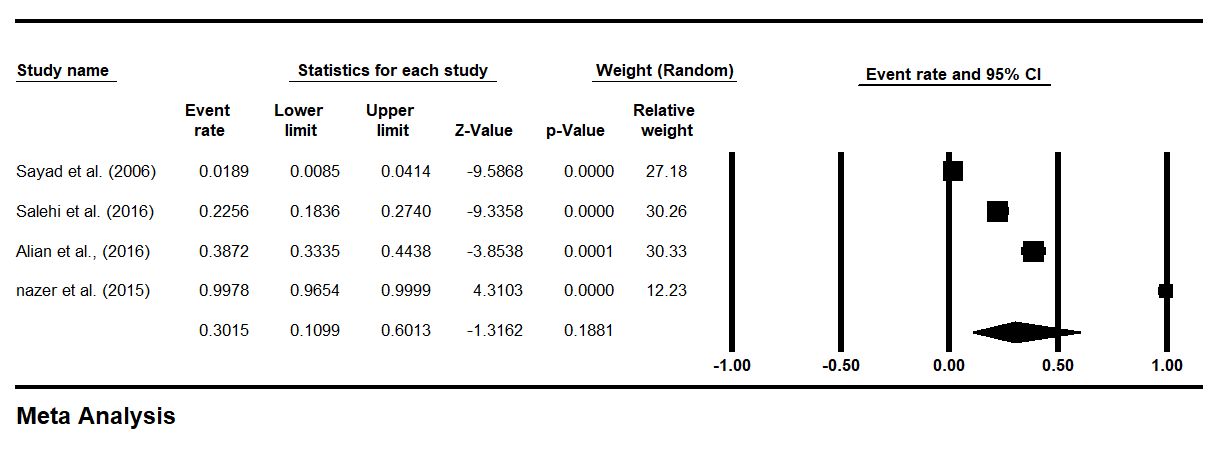

Supplement: S8 Fig — (TIF) [file pone.0223335.s012.tif]

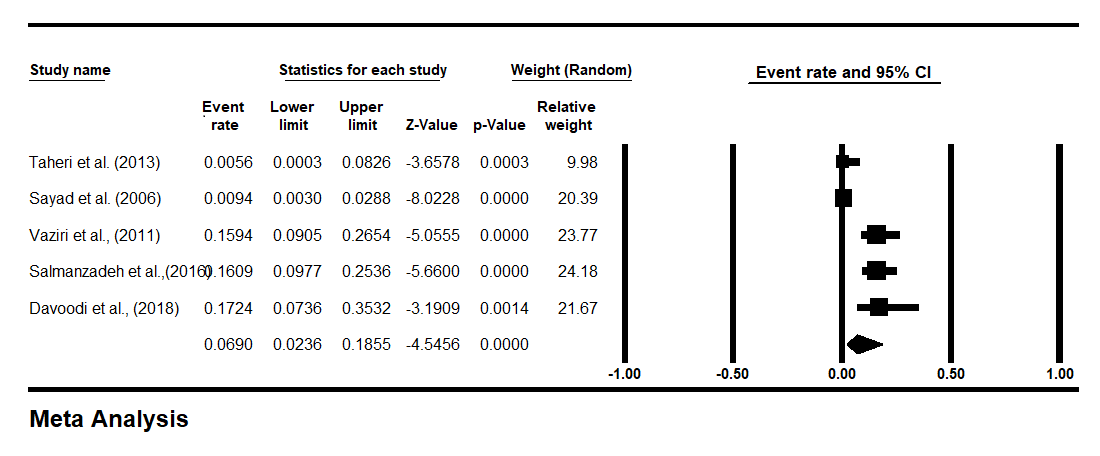

Supplement: S9 Fig — (TIF) [file pone.0223335.s013.tif]

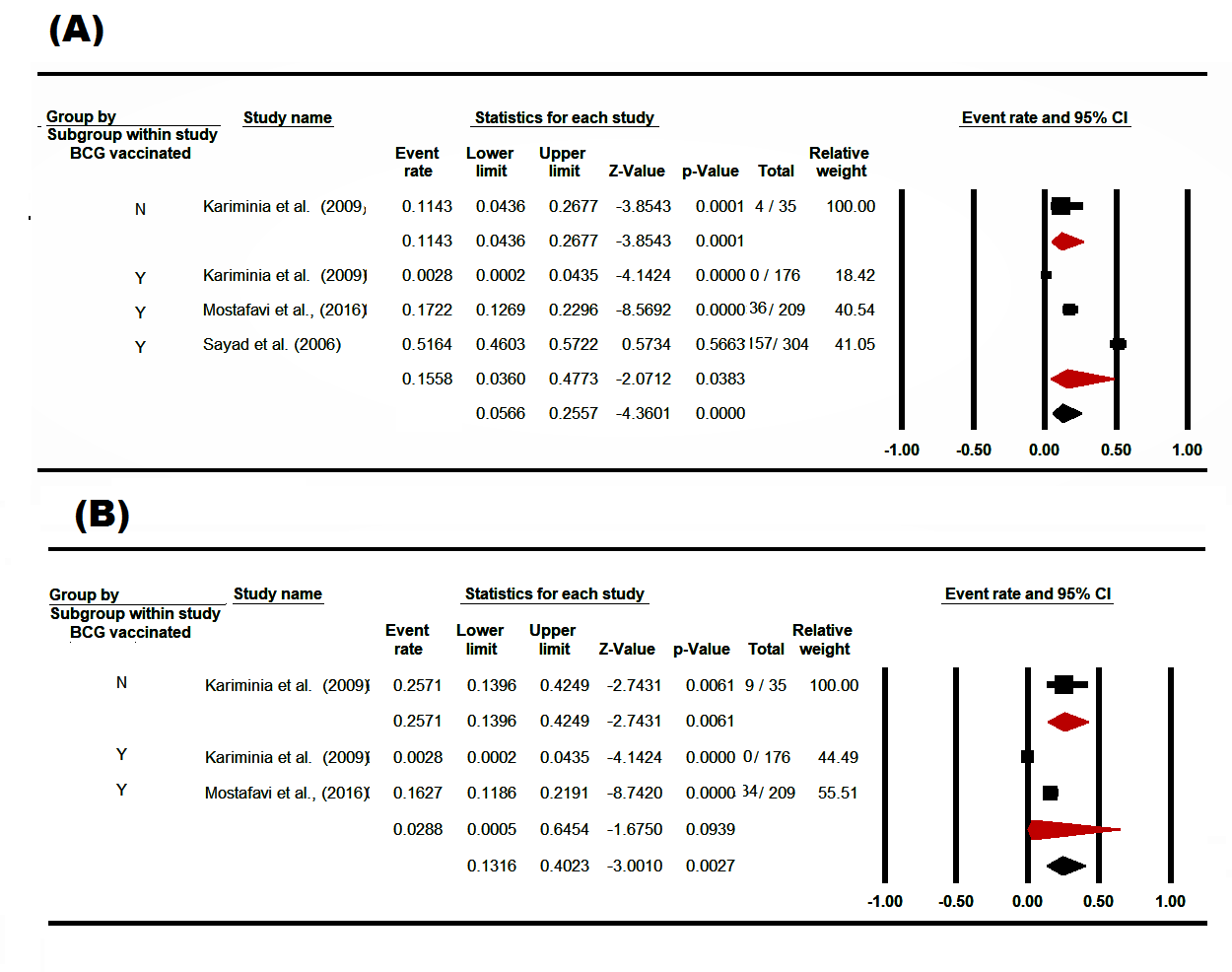

Supplement: S10 Fig — (TIF) [file pone.0223335.s014.tif]

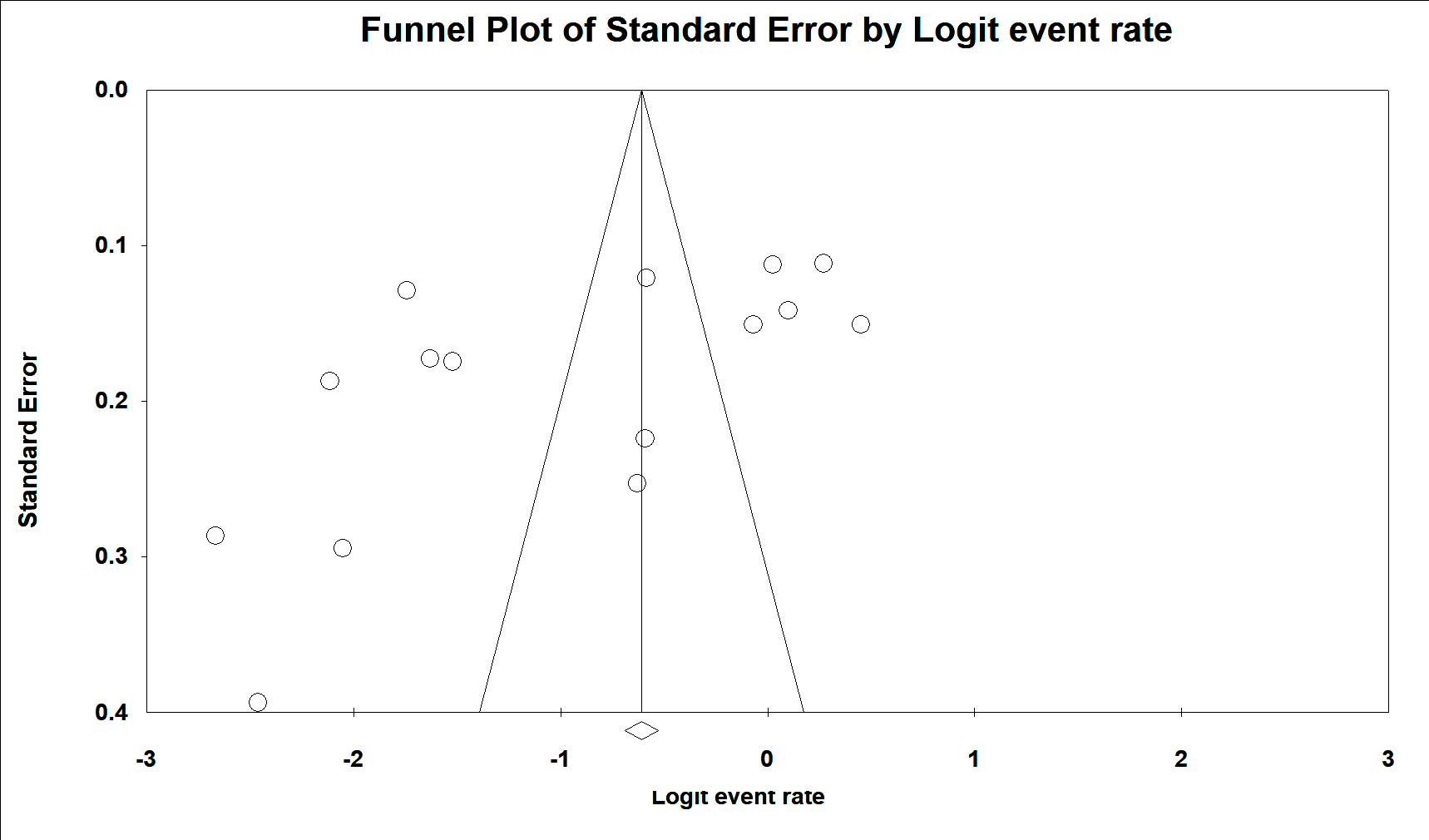

Supplement: S11 Fig — (TIF) [file pone.0223335.s015.tif]
