# Supplementary material for: Is latent tuberculosis infection challenging in Iranian health care workers? A systematic review and meta-analysis
Source: PLoS One. 2019 Oct 3;14(10):e0223335. doi: 10.1371/journal.pone.0223335 (PMC6776393; doi:10.1371/journal.pone.0223335)
Supplement: Attachment — Submitted filename: PONE-D-19-18112_reviewer.pdf [file pone.0223335.s016.pdf]

PLOS ONE

Is latent tuberculosis infection challenging in Iranian health care workers? A systematic review and meta-analysis

--Manuscript Draft--

**Manuscript Number:**	PONE-D-19-18112
**Article Type:**	Research Article
**Full Title:**	Is latent tuberculosis infection challenging in Iranian health care workers? A systematic review and meta-analysis
**Short Title:**	LT BI among HCWs in Iran
**Corresponding Author:**	Alireza Jafari<br>Inflammatory Lung Disease Research Center<br>Rasht, IRAN, ISLAMIC REPUBLIC OF
**Keywords:**	Latent Tuberculosis; Health Personnel; Iran
**Abstract:**	BaBackground: Latent tuberculosis infection (LTBI) has been changed to one of the challenges of health care workers (HCWs) in low middle-income countries.<br>Method: Search strategies that were lead through Persian (national) databases include SID, Barakat knowledge network system; Irandoc, Magiran; Iranian national library. The international database was Web of science, Scopus, PubMed/MEDLINE, OVID, EMBASE, the Cochrane library, and Google Scholar search engine. The Persian and the English languages were used as the filter in national and international databases, respectively. Searching was done through MeSH terms. The search terms were conducted till January 01, 2019.<br>Results: The prevalence of LTBI in Iranian HCWs, based on the PPD test was (27.13% [CI95%: 18.64-37.7]). The highest prevalence of LTBI in HCWs were estimated (41.4 % [CL95%: 25.4-59.5] in the north, and (33.8% [CI95%: 21.1-49.3]) in the west of Iran. The lowest prevalence of LTBI was evaluated (18.2% [CI95%: 3.4-58.2]) in the south of Iran. The prevalence of LTBI in HCWs who had work-experience more than 20 years old were estimated (20.49% [CI95%: 11-34.97]). In the PPD test, the prevalence of LTBI in HCWs who had received the Bacille Calmette–Guérin (BCG) was estimated (15% [CI 95%: 3.6-47.73]). While, in the QFT, the prevalence of LTBI in HCWs in non-vaccinated was estimated (25.71% [CI95%: 13.96-42.49]).<br>Conclusions: This meta-analysis shows the highest prevalence of LTBI in HCWs in the north and the west of Iran due to neighboring countries like Azerbaijan and Iraq, respectively. Hence, Iranian HCWs do not fully understand the isolation and personal protection. We also found that BCG was not able to protect Iranian HCWs from TB infections, completely.
**Order of Authors:**	Mohammad Hossein YektaKooshali
	Farahnaz Movahedzadeh
	Ali Alavi Foumani
	Hoda Sabati
	Alireza Jafari
**Additional Information:**	
**Question**	**Response**
**Financial Disclosure**<br><br>Enter a financial disclosure statement that describes the sources of funding for the work included in this submission. Review the submission guidelines for detailed requirements. View published research articles from *PLOS ONE* for specific	The author(s) received no specific funding for this work.

Powered by Editorial Manager® and ProduXion Manager® from Aries Systems Corporation

examples.<br><br>This statement is required for submission and **will appear in the published article** if the submission is accepted. Please make sure it is accurate.<br><br>**Unfunded studies**<br>Enter: *The author(s) received no specific funding for this work.*<br><br>**Funded studies**<br>Enter a statement with the following details:<br>• Initials of the authors who received each award<br>• Grant numbers awarded to each author<br>• The full name of each funder<br>• URL of each funder website<br>• Did the sponsors or funders play any role in the study design, data collection and analysis, decision to publish, or preparation of the manuscript?<br>• **NO** - Include this sentence at the end of your statement: *The funders had no role in study design, data collection and analysis, decision to publish, or preparation of the manuscript.*<br>• **YES** - Specify the role(s) played.<br><br>* typeset	
**Competing Interests**<br><br>Use the instructions below to enter a competing interest statement for this submission. On behalf of all authors, disclose any competing interests that could be perceived to bias this work—acknowledging all financial support and any other relevant financial or non-financial competing interests.<br><br>This statement **will appear in the published article** if the submission is accepted. Please make sure it is accurate. View published research articles from *PLOS ONE* for specific examples.	The authors have declared that no competing interests exist.

**NO authors have competing interests**<br><br>Enter: *The authors have declared that no competing interests exist.*<br><br>**Authors with competing interests**<br><br>Enter competing interest details beginning with this statement:<br><br>*I have read the journal's policy and the authors of this manuscript have the following competing interests: [insert competing interests here]*<br><br>* typeset	
**Ethics Statement**<br><br>Enter an ethics statement for this submission. This statement is required if the study involved:<br><br>• Human participants<br>• Human specimens or tissue<br>• Vertebrate animals or cephalopods<br>• Vertebrate embryos or tissues<br>• Field research<br><br>Write "N/A" if the submission does not require an ethics statement.<br><br>General guidance is provided below. Consult the submission guidelines for detailed instructions. **Make sure that all information entered here is included in the Methods section of the manuscript.**	N/A

Powered by Editorial Manager® and ProduXion Manager® from Aries Systems Corporation

**Format for specific study types**<br><br>**Human Subject Research (involving human participants and/or tissue)**<br>• Give the name of the institutional review board or ethics committee that approved the study<br>• Include the approval number and/or a statement indicating approval of this research<br>• Indicate the form of consent obtained (written/oral) or the reason that consent was not obtained (e.g. the data were analyzed anonymously)<br><br>**Animal Research (involving vertebrate animals, embryos or tissues)**<br>• Provide the name of the Institutional Animal Care and Use Committee (IACUC) or other relevant ethics board that reviewed the study protocol, and indicate whether they approved this research or granted a formal waiver of ethical approval<br>• Include an approval number if one was obtained<br>• If the study involved *non-human primates*, add *additional details* about animal welfare and steps taken to ameliorate suffering<br>• If anesthesia, euthanasia, or any kind of animal sacrifice is part of the study, include briefly which substances and/or methods were applied<br><br>**Field Research**<br><br>Include the following details if this study involves the collection of plant, animal, or other materials from a natural setting:<br>• Field permit number<br>• Name of the institution or relevant body that granted permission	
**Data Availability**<br><br>Authors are required to make all data underlying the findings described fully available, without restriction, and from the time of publication. PLOS allows rare exceptions to address legal and ethical concerns. See the PLOS Data Policy and FAQ for detailed information.	Yes - all data are fully available without restriction

A Data Availability Statement describing where the data can be found is required at submission. Your answers to this question constitute the Data Availability Statement and **will be published in the article**, if accepted.<br><br>**Important:** Stating 'data available on request from the author' is not sufficient. If your data are only available upon request, select 'No' for the first question and explain your exceptional situation in the text box.<br><br>Do the authors confirm that all data underlying the findings described in their manuscript are fully available without restriction?	
**Describe where the data may be found in full sentences. If you are copying our sample text, replace any instances of XXX with the appropriate details.**<br><br>• If the data are **held or will be held in a public repository**, include URLs, accession numbers or DOIs. If this information will only be available after acceptance, indicate this by ticking the box below. For example: *All XXX files are available from the XXX database (accession number(s) XXX, XXX.).*<br>• If the data are all contained **within the manuscript and/or Supporting Information files**, enter the following: *All relevant data are within the manuscript and its Supporting Information files.*<br>• If neither of these applies but you are able to provide **details of access elsewhere**, with or without limitations, please do so. For example:<br><br>*Data cannot be shared publicly because of [XXX]. Data are available from the XXX Institutional Data Access / Ethics Committee (contact via XXX) for researchers who meet the criteria for access to confidential data.*<br><br>*The data underlying the results presented in the study are available from (include the name of the third party*	All relevant data are within the manuscript and its Supporting Information files.

and contact information or URL).<br>• This text is appropriate if the data are owned by a third party and authors do not have permission to share the data.<br><br>* typeset	
Additional data availability information:	

Powered by Editorial Manager® and ProduXion Manager® from Aries Systems Corporation

Main manuscript Click here to access/download;Manuscript;Main manuscript (Plos One) 980407 edited.docx

Is latent tuberculosis infection challenging in Iranian health care workers? A systematic review and meta-analysis

Running title: LT BI among HCWs in Iran

Mohammad Hossein YektaKooshali[1, 2], Farahnaz Movahedzadeh[3, 4], Ali Alavi Foumani[2], Hoda Sabati[5], Alireza Jafar[2, 6*]

[1] Research Assistant, Student Research Committee, School of nursing, Midwifery and Paramedicine, Guilan University of Medical Sciences, Rasht, Iran

[2] Inflammatory Lung Diseases Research Center, Department of Internal Medicine, Razi Hospital, School of Medicine, Guilan University of Medical Sciences, Rasht, Iran

[3] Institute for Tuberculosis Research, College of Pharmacy, University of Illinois at Chicago, Chicago, Illinois, USA

[4] Department of Medicinal Chemistry and Pharmacognosy, College of Pharmacy University of Illinois at Chicago, Chicago, Illinois, USA

[5] Biotechnology and Biological Science Research Center, Faculty of Science, Shahid Chamran University of Ahvaz, Iran

[6] Urology Research Center, Department of Internal Medicine, Razi Hospital, School of Medicine, Guilan University of Medical Sciences, Rasht, Iran

Corresponding author:

Dr. Alireza Jafari

Inflammatory Lung Disease Research Center, Department of Internal Medicine, Razi Hospital, School of Medicine, Guilan University of Medical Sciences, Rasht, Iran

Urology Research Center, Department of Internal Medicine, Razi Hospital, School of Medicine, Guilan University of Medical Sciences, Rasht, Iran

Address: No. 11, Sardar Jangal Road, Razi Hospital, Rasht, Iran

Tel: +98 912 346 2738 ; Fax: +98 13 33542460

E-mail: Dr.alireza.jafariii@gmail.com

Abstract:

Background: Latent tuberculosis infection (LTBI) has been changed to one of the challenges of health care workers (HCWs) in low middle-income countries.

Method: Search strategies that were lead through Persian)national(databases include SID, Barakat knowledge network system; Irandoc, Magiran; Iranian national library. The international database was Web of science, Scopus, PubMed/MEDLINE, OVID, EMBASE, the Cochrane library, and Google Scholar search engine. The Persian and the English languages were used as the filter in national and international databases, respectively. Searching was done through MeSH terms. The search terms were conducted till January 01, 2019.

Results: The prevalence of LTBI in Iranian HCWs, based on the PPD test was (27.13% [CI95%: 18.64-37.7]). The highest prevalence of LTBI in HCWs were estimated (41.4 % [CL95%: 25.4-59.5] in the north, and (33.8% [CI95%: 21.1-49.3]) in the west of Iran. The lowest prevalence of LTBI was evaluated (18.2% [CI95%: 3.4-58.2]) in the south of Iran. The prevalence of LTBI in HCWs who had work-experience more than 20 years old were estimated (20.49% [CI95%: 11-34.97]). In the PPD test, the prevalence of LTBI in HCWs who had received the Bacille Calmette–Guérin (BCG) was estimated (15% [CI 95%: 3.6-47.73]). While, in the QFT, the prevalence of LTBI in HCWs in non-vaccinated was estimated (25.71% [CI95%: 13.96-42.49]).

Conclusions: This meta-analysis shows the highest prevalence of LTBI in HCWs in the north and the west of Iran due to neighboring countries like Azerbaijan and Iraq, respectively. Hence, Iranian HCWs do not fully understand the isolation and personal protection. We also found that BCG was not able to protect Iranian HCWs from TB infections, completely.

Keywords: Latent Tuberculosis; Health Personnel; Iran

1. Introduction:

Latent tuberculosis infection (LTBI) is an immune response to *Mycobacterium tuberculosis* (Mtb) antigens without symptoms of active tuberculosis (TB) [1]. Mtb is able to colonize inside the alveolar macrophages and finally form granuloma. Mtb is ingested by phagocytosis by resident alveolar macrophages and tissue dendritic cells (DC) [2, 3]. The immune cells contribute and the pathological mark of TB, the granuloma, is formed. In the granuloma, macrophages differentiate into epithelial cells or foamy macrophages, or fuse to form giant cells, and become surrounded by lymphocytes, fibroblasts and extracellular matrix proteins. In such conditions, the Mtb will be surviving until the granuloma fails due to immunosuppression [4, 5]. Mtb use the granuloma as they are effective at initial infection level since they recruit new macrophages to allow the spread of infection between host cells [6]. At this stage, the LTBI is formed in the patient's body.

[7]. There are several reports of LTBI outbreaks in Iran; however, the highest prevalence of LTBI has been reported to be 82% in Sistan-Baluchistan province [8]. The risk of tuberculosis in health care workers (HCWs) is estimated to be twice as high in the general population, in high-income countries, and five times higher than the general population in countries with a low and middle income [9, 10].

In addition, one of the challenges in many countries is the transfer of tuberculosis from patients admitted to the hospital to HCWs [10]. Most importantly, the transfer of resistant Mycobacterium tuberculosis strains from admitted patients to HCWs has increased the importance of the subject [11].

According to the findings, direct exposure to HCWs in patients with tuberculosis, direct contact with phlegm specimens and blood products of suspected tuberculosis patients, and long hours of work in high-risk places increases the risk of tuberculosis infection [12, 13]. This means that direct contact is one of the most important and worrisome factor in the

transmission of tuberculosis to HCWs [10-12, 14]. Work experience, age [15], occupational status [16], the use of personal protective equipment, ventilation, hospital Infection Control Unit and infection control in isolation rooms can affect LTBI outbreaks in HCWs [9-12]., T [17]. To diagnose LTBI, the Mantoux tuberculin skin test (TST) and QuantiFERON-TB Gold (QFT) are used [18]. Studies have shown that QFT has a higher sensitivity and specificity in detecting LTBI [19, 20]. However, some researchers believe that QFT is not superior to TST in detecting LTBI [21-23].

The early detection of LTBI in controlling, treating and preventing Mtb is a key element in patients. So that preventive treatment can reduce the risk of active tuberculosis in patients by up to 90% [24]. So far, systematic review and meta-analysis has not been conducted to evaluate the prevalence and risk factors of LTBI among HCWs in Iran. In Iran, the Centers for Disease Control and Prevention (CDC) do not control the Mtb as a regular program, however, reports of LTBI outbreaks in HCWs attracts a high controversy [25]. Due to the highest level of evidence and an essential role in evidence-based decision-making of meta-analysis studies [26, 27], this study estimated the prevalence and risk factors of LTBI among HCWs in Iran which can have vital information for policy-makers and planning at the country level.

2. Materials and methods

2.1. Study Protocol

This is the first study that was conducted based on the meta-analysis of observational studies according to epidemiology guidelines [27], and the PRISMA (Preferred Reporting Items for Systematic Reviews and Meta-Analyses) statement (S1 File) [28]. The study was achieved based on five steps; design and search strategy; collecting original articles; evaluating inclusion and exclusion criteria, and finally qualitative evaluation and statistical analysis of data. Two independent researchers)MH. YK& A.J(evaluated the data. The disagreements were solved by consensus between the team, and a bacteriologist (H.S.E). The review protocol was registered in PROSPERO :International Prospective Register of Systematic Reviews) https://www.crd.york.ac.uk/PROSPERO/) Identifier: CRD42018117682 [29, 30] (S2 File).

2.2. Search strategy

In order to maximize its sensitivity, search strategy was six lead through Persian (national) databases, including scientific information database (SID))http://www.sid.ir/), Barakat knowledge network system)http://health.barakatkns.com), Iranian research institute for information science and technology)IranDoc( )https://irandoc.ac.ir), Magiran)http://www.magiran.com), Iranian national library)http://www.nlai.ir/). The international databases, including web of science, Scopus, PubMed/MEDLINE, OVID, EMBASE, the Cochrane Library)Cochrane Database of Systematic Reviews(,and Google Scholar search engine. he Persian and the English T languages were used as the filter in national and international databases, respectively. The search terms were adapted to international databases. To search a combination of words, Boolean operators) AND& OR(were used. hing was done through medical Searc subject heading)MeSH(terms. The search terms were conducted without any time limitation

till January 01, 2019. The authors were then independently analyzed the manuscript contained in the title and abstract. For instance, PubMed search formula was provided in the appendix.

2.3. Inclusion and exclusion criteria

2.3.1. Inclusion criteria based on PICO (related to Evidence-Based Medicine) [31, 32]

(1) **P**opulation: This study was been concentrated on the population of HCWs with LTBI who were residents in the geographic regions of Northern, Southern, Eastern, Western, center, and capital city of Iran. ;)2(**I**ntervention: The exposure were the laboratory tests (Interferon-gamma (IFN-γ) release assay (IGRA), and tuberculin skin tests)TST((of which confirmed LTBI among HCWs in Iran. ;)3(**C**omparison: A population of HCWs who did not have signs of active TB disease and did not feel illness.; (4) **O**utcome: Estimate the overall prevalence and risk factors of LTBI infection among HCWs in Iran.

2.3.2. Exclusion criteria

Review articles, letters, editorial, case reports, conference papers, and comments were excluded. The studies of which did not have a focus on the prevalence of LTBI in Iranian HCWs, duplicated papers, non-English full papers, non-Persian full papers, and non-assessable full-text papers were excluded. Likewise, the populations other than Iranian HCWs were excluded.

2.4. Latent TB detection criteria

2.4.1. The Mantoux tuberculin skin test (TST)

To the Mantoux tuberculin skin test (TST), purified protein derivative (0.1 Ml) is used [33-35], and the induration at TST site is measured 72 hours later. TST reaction of ≥ 5 mm of induration is classified as negative but is considered as positive in patients receiving corticosteroid or patients with Acquired Immunodeficiency Syndrome (AIDS), diabetes

mellitus, lymphoma, and leukemia. The induration of ≥10 mm is classified positive in; recent immigrants (< 5 years) from high-prevalence countries; residents and employees of high-risk congregate settings; mycobacteriology laboratory personnel; persons with clinical conditions that place them at high risk. The induration of ≥15 mm is considered positive in any person, including persons with no known risk factors for TB. Two-step testing methods were used for health care workers and nursing home residents [33-35].

2.4.2. Interferon-gamma release assays

Interferon-gamma release assays (IGRAs) show how the immune system reacts to the Mycobacteria that cause TB [36]. The IGRA has been approved by the U.S. Food and Drug Administration (FDA). Positive IGRA means that the person has been infected with TB bacteria. Negative IGRA means that the person's blood did not react to the test and that latent TB infection or TB disease is not likely. IGRA is the preferred method of TB infection testing for people who have received the Bacille Calmette–Guérin (BCG) [30, 36-39].

2.5. Selection of studies

During the selection stage, duplicated studies were removed by the EndNote™ software Ver. X9 (Clarivate Analytics company). In the skimming and screening stage, co-authors, journals, and publishing years were evaluated by two experts based on inclusion and exclusion criteria (the eligibility stage), independently. The disagreements between the two were resolved through an expert bacteriologist (Figure 1).

Fig 1. A flow diagram (Stacked Venn) following the PRISMA (Depicted by MH-YK).

2.6. Quality appraisal

In this stage, the irrelevant studies were excluded, and then the quality of each study was evaluated. To quality appraisal, the Newcastle-Ottawa Scale (NOS) checklist (S3 File)[40] was applied whichdetermined the quality of these studies based on three levels of scoring.

The score of five or less defined a poor quality study; the score of five or six distinguished as the medium quality study, and the score of seven or eight determined as the high-quality study. Finally, the medium to high-quality studies were included in the data analysis (Fig 1).

Fig 1. A flow diagram (Stacked Venn) following the PRISMA (Depicted by MH-YK).

2.7. Data extraction

The enter terms were author's names, province, geographical regions, year of publishing, sample size, age ,gender ,history of BCG ,history of exposure with tuberculosis ,history of tuberculosis disease ,laboratory diagnosis tests, job experience, duration of employment, workplaces, single-step or two-step TST, and history of hospitalization. The author's name, institution, and the journal name were blinded, and then data was extracted through two researchers (MH.YK & A.J), independently. Only if necessary, the additional information/raw data was collected by phone call, mailing, or fax.

2.8. Statistical analysis

The prevalence of LTBI in HCWs was considered as a binomial distribution probability, and the variance was calculated by a binomial distribution. To evaluate its heterogeneity, the Cochran test (Q) and I^2 index were used[1, 41, 42]. The subgroup analysis was performed based on province, single-step or two-step TST, laboratory diagnosis tests, job, gender, history of TB disease, history of TB exposure, history of BCG, and geographical regions. Sensitivity analysis was also achieved to evaluate the impact of each study, based on the results of the overall prevalence of LTBI in Iranian HCWs. The Begg's test and Egger's test were carried out using a funnel plot to examine publication bias. Data analysis was examined by the Comprehensive Meta-Analysis Ver .2)Englewood ,NJ 07631, USA((,and level of significance was considered as $p<0.05$.

3. Results:

3.1. Study characteristics and methodological quality

In the primary search of study, 421 studies were found. After skimming and screening, 20 (4.75%) studies were eligible according to inclusions and exclusions criteria [43-62]. The total sample size was calculated 6453 Iranian HCWs (Fig 1) (S1 Table.(

Fig 1. A flow diagram (Stacked Venn) following the PRISMA (Depicted by MH-YK).

3.2. The overall prevalence LTBI in HCWs

The prevalence of LTBI in HCWs, based on the PPD test (48 hours) was (27.13% [CI95%: 18.64-37.7]) (Fig 2), and based on the QFT test was (16.92% [CI95%: 9.7-27.84]) (Fig 3). The prevalence of LTBI was estimated (12.11% [CI95%: 4.53-28.57]) in Iranian HCWs who had negative TST reaction (48 hours) in the first week (Fig 4). The prevalence of induration at TST site (48 h) was estimated <4 mm in (43.74% [CI 95%: 28.19-60.63]), 5-9 mm in (17.52% [CI 95%: 9.73-29.5]), 10-15 mm in (14.55% [CI 95%: 8.87-22.93]) and >15 mm in (13.4% [CI 95%: 8.59-20.31]) (S1 Fig).

Fig 2. The prevalence subgroup analysis (Forest plot - Random effect model) based on TST/PPD induration diameter (48 hrs.) in HCWs with LTBI.

Fig 3. The prevalence subgroup analysis (Forest plot - Random effect model) based on QFT in HCWs with LTBI.

Fig 4. The prevalence subgroup analysis (Forest plot - Random effect model) based on TST/PPD induration diameter after one week in HCWs with LTBI.

3.3. The prevalence of LTBI in Iranian HCWs based on geographical region of Iran

The highest prevalence of LTBI in HCWs was estimated (41.4 % [CL95%: 25.4-59.5] in the north, and (33.8% [CI95%: 21.1-49.3]) in the west of Iran. The lowest prevalence of LTBI was evaluated (18.2% [CI95%: 3.4-58.2]) in the south of Iran. These results showed a

significant relationship between LTBI prevalence in HCWs and the geographic location in Iran (p <0.0001) (S2 Fig) (Fig 5).

Fig 5. Distribution of LTBI in Iranian HCWs based on geographical classification (Random effect model).

3.4. Sensitivity analysis and cumulative meta-analysis

Sensitivity analysis of prevalence of LTBI in Iranian HCWs was estimated with a 95% confidence interval. It showed that there is no significant effect on the overall prevalence of LTBI in Iranian HCWs (Fig 6). The overall prevalence of LTBI in Iranian HCWs based on the publication year was estimated by cumulative meta-analysis and represented in (S3 Fig).

Fig 6. Sensitivity analysis to prevalence of LTBI in Iranian HCWs (one study removed test)

3.5. Meta-regression

The prevalence of publishing manuscripts about identification of LTBI in HCWs by the PPD test (48 hours), has decreased in Iran. There was however no significant relationship. (Mixed effects regression (Method of moments); Slope = -0.1898(SE = 0.068, (95% CI: -0.323– -0.056)), Intercept = 381.14 (SE = 137.43, (95% CI: 111.78–650.5)), P = 0.10653) (Fig 7).

Fig 7. Meta-regression of LTBI in Iranian HCWs according to publishing year of studies (method of moments).

3.6. The prevalence of LTBI in HCWs based on term of employment

The prevalence of LTBI in Iranian HCWs with more than 10 years old work-experience was evaluated 51%. The prevalence of LTBI in HCWs with less than 10 years old work-experience was estimated 29.30% in the PPD test. The prevalence of LTBI in HCWs with more than 20 years old work-experience was calculated (20.49% [CI95%: 11-34.97]), which

showed a significant relationship between the term of employment in QFT (P <0.0001) (S4 Fig).

3.7. The prevalence of LTBI in Iranian HCWs based on occupation and wards

The prevalence of LTBI in assistant nurses was estimated 45.76% [CI 95%: 33.51-58.55], in physicians was evaluated 44.99% [CI95%: 33.37-57.17], in ward nurses was calculated 39.4% [CI95%: 17.63-66.39], and in service workers was estimated 36.43% [CI95%: 19.51-57.53] based on PPD test. The prevalence of LTBI in both nurses and TB service workers was higher than other occupations based on QFT (Fig 8).

Fig 8. The prevalence subgroup analysis of occupational (Forest plot - Random effect model) based on PPD (A), and QFT (B) in HCWs with LTBI.

The prevalence of LTBI in the infectious ward was estimated (52.09% [CI95%: 43.92-60.14]), and in the internal ward was evaluated (50% [CI95%: 34.22-65.78]. The lowest prevalence of LTBI was estimated in the infectious wards based on QFT. There was a significant relationship between the prevalence of LTBI in HCWs, and hospital wards (p <0.0001) (Fig 9).

Fig 9. The prevalence subgroup analysis of ward (Forest plot - Random effect model) base on PPD (A), and QFT (B) in HCWs with LTBI.

3.8. The prevalence of LTBI in HCWs based on gender and age

The prevalence of LTBI was estimated at 42.16% [CI95%: 26.41-59.69] in male Iranian HCWs based on the PPD test. The prevalence of LTBI in Iranian HCWs and type of gender based on PPD test (P <0.051). In QFT, however, a significant relationship was showed between the prevalence of LTBI in HCWs, and the gender (P <0.0001) (S5 Fig).

The prevalence of LTBI in HCWs who were more than 40 years old was estimated 44% [CI 95%: 26.47-63.16] in the PPD test.

The prevalence of LTBI in Iranian HCWs aged 30 years old was estimated 22.52% [CI95 %: 3.7-68.34] in the QFT. In both PPD test and QFT, it was evaluated that there was no significant relationship between the prevalence of LTBI in HCWs, and age of HCWs (P <0.0001) (S6 Fig).

3.9. The prevalence of LTBI in HCWs based on the history of tuberculosis contact and the tuberculosis clinical symptoms

The results showed that 30.15% [CI 95%: 11-60.13] of Iranian HCWs directly contacted to patients with tuberculosis. The results also showed that 6.9% [CI 95%: 2.36-18.55] of Iranian HCWs had active tuberculosis symptoms (S7-8 Fig).

3.10. The prevalence of LTBI in HCWs based on the "BCG"

The prevalence of LTBI in Iranian HCWs who received the BCG was estimated (15% [CI 95%: 3.6-47.73]) based on the PPD test. While the prevalence of LTBI in Iranian HCWs who did not receive the BCG was estimated at 25.71% [CI95%: 13.96-42.49] based on the QFT. In both PPD, and QFT, there was a significant relationship between those who did and those who did not receive the vaccine (S9 Fig).

3.11. Publication bias

The publication bias in this study was evaluated by Begg's and Egger's tests. The publication bias by Begg's test was calculated 0.06, and the Egger's test was calculated 0.028. The probability of the publication bias in this study was significant (S10 Fig).

4. Discussion:

This study is the first systematic review and meta-analysis of latent tuberculosis outbreak (LTBI) been carried out in health care workers (HCWs) in Iran. According to results of the current meta-analysis, the prevalence of LTBI in HCWs in Iran is estimated at 27.1% [1]. Among the low- and middle-income countries, the prevalence of LTBI in Kenya [63], Zimbabwe [64], Russia [65], Brazil [66], Vietnam [67], Rwanda [68], China [69] and South Africa [70] has been higher than in Iran [1]. The prevalence of LTBI in HCWs Italy [71], Norway [72] and India [73] is reported to be equal to or less than Iran. Iran is a TB endemic country [25] and the treatment of latent tuberculosis is usually done by using a single medicine and only in high risk groups [74]. While in high-income countries, screening of pulmonary and lab staffs is recommended annually [75]. Also, based on the prevalence of LTBI in Iranian HCWs, it could be seen that Iranian HCWs training is not sufficient in confronting a patient with tuberculosis [25]. Hence, Iranian HCWs do not fully understand isolation and personal protection [25].

According to the results of meta-analysis, the lowest prevalence of LTBI in HCWs was in southern Iran (18.2%). The highest prevalence of LTBI in HCWs was reported in northern and western Iran. The high prevalence of LTBI in HCWs may be due to neighboring Azerbaijan and Iraq [76].

Azerbaijan which is listed on the high burden countries has high prevalence of multidrug resistance MTB [76-78]. In fact, the northern neighbors of Iran, such as Kazakhstan, Azerbaijan, are among the high burden countries with a high prevalence of multi-drug resistant tuberculosis [80].

On the other hands, the name of the country's western neighbor of Iran –Iraq- is not listed on the high burden countries [76-78] but according to reports from Ministry of Health - Iran Center for Medical Education and Treatment, Infectious Disease Control Center- the Iraqi

state may have become a high-risk source for tuberculosis after undergoing its recent crisis [79, 80].

The current study showed that 15.5% of the HCWs used before the BCG with at least a positive PPD test. According to studies, BCG does not protect adults from getting infected with tuberculosis, so the positive results of tuberculin testing in people vaccinated with BCG will be considered as a latent infection [81]. In other words, previous vaccination with BCG prevents tuberculin testing [82]. This may be due to a false positive reaction in PPD [25]. Some HCWs may respond to skin tests without being infected with mycobacterium [83]. The reason for these false-positive reactions may be due to contamination with non-tuberculosis mycobacterium, previous BCG, poor test performance or inappropriate interpretation of the test [83].

5. Limitations

Information about this meta-analysis was extracted from data published in Iranian databases as there was no access to the actual information of the control center of the Ministry of Health and Medical Education, so the exact prevalence of LTBI in HCWs could not be calculated.

Selection bias is able to limit the generalization of these findings because the type of bacteria strains in a country could be different with the other countries and could be related to descent diversities.

On the other hands, patients may not respond to skin test tuberculosis, even if they are infected with *Mycobacterium*. It may be due to skin allergies, recent infections (recent contact for 8 to 10 weeks), chronic infection, recent vaccinations with live viruses, advanced tuberculosis, some viral diseases (measles and bile), misdiagnosis skin or incorrect interpretation of the reaction. Patients may also respond to skin tests, even without being infected with *Mycobacterium*. The reason for these reactions may be due to contamination

with non-tuberculosis *Mycobacterium*, previous BCG, inappropriate test run or inappropriate interpretation of the test.

Despite the fact that the CDC updates the guidelines for the prevention and transmission of *M. tuberculosis* in health-care settings annually, the protocol for HCWs in Iran has not yet been prepared. Also, workshops could be developed to train tuberculosis prevention and self-care HCWs in the western regions of the Iran.

National databases are not sensitive to operators “AND” and “OR” to search for the combinations. Also, some databases were not fully accessible because of using Guilan University of Medical Sciences’ - Iran Ministry of Health & Medical Education- VPN.

6. Conclusion

This meta-analysis showed the prevalence of LTBI in HCWs in Iran and estimated at 27.1%. The prevalence of LTBI in HCWs of Italy, Norway and India is reported to be equal to or less than HCWs of Iran. On the other hand, the highest prevalence of LTBI in HCWs in the north and the west of Iran may due to neighboring with Azerbaijan and Iraq which has become a high-risk source for tuberculosis by overcoming its recent years of crisis. Meanwhile, HCWs training is not sufficient in confronting a patient with tuberculosis, in Iran. Hence, Iranian HCWs do not fully understand the isolation and personal protection. We also found that BCG was not able to protect Iranian HCWs from TB infectious, completely.

7. Competing Interests

The authors declare that they have no competing interests.

8. Acknowledgment

We would like to thank the vice chancellor of research and technology, Guilan University of Medical Sciences (ID: IR.GUMS.REC.1397.513).

9. Author Contributions

Conceptualization: Alireza Jafari, Mohammad Hossein YektaKooshali

Data curation: Mohammad Hossein YektaKooshali

Formal analysis: Mohammad Hossein YektaKooshali

Funding acquisition: Mohammad Hossein YektaKooshali

Investigation: Alireza Jafari, Farahnaz Movahedzadeh, Ali Alavi Foumani

Methodology: Mohammad Hossein YektaKooshali

Project administration: Alireza Jafari, Mohammad Hossein YektaKooshali

Resources: Alireza Jafari

Software: Mohammad Hossein YektaKooshali

Supervision: Farahnaz Movahedzadeh, Ali Alavi Foumani

Validation: Mohammad Hossein YektaKooshali

Visualization: Mohammad Hossein YektaKooshali

Writing ± original draft: Alireza Jafari, Mohammad Hossein YektaKooshali, Hoda Sabati

Writing ± review & editing: Mohammad Hossein YektaKooshali, Alireza Jafari, Hoda Sabati

10. References

1. Apriani L, McAllister S, Sharples K, Alisjahbana B, Ruslami R, Hill PC, et al. Latent tuberculosis infection in healthcare workers in low- and middle-income countries: an updated systematic review. The European respiratory journal. 2019;53(4). Epub 2019/02/23. doi: 10.1183/13993003.01789-2018. PubMed PMID: 30792341.
2. Korbel DS, Schneider BE, Schaible UE. Innate immunity in tuberculosis: myths and truth. Microbes Infect. 2008;10(9):995-1004. Epub 2008/09/03. doi: 10.1016/j.micinf.2008.07.039. PubMed PMID: 18762264.
3. North RJ, Jung YJ. Immunity to tuberculosis. Annu Rev Immunol. 2004;22:599-623. Epub 2004/03/23. doi: 10.1146/annurev.immunol.22.012703.104635. PubMed PMID: 15032590.
4. Russell DG. Who puts the tubercle in tuberculosis? Nat Rev Microbiol. 2007;5(1):39-47. Epub 2006/12/13. doi: 10.1038/nrmicro1538. PubMed PMID: 17160001.
5. De Chastellier C. The many niches and strategies used by pathogenic mycobacteria for survival within host macrophages. Immunobiology. 2009;214(7):526-42.
6. Davis JM, Ramakrishnan L. The role of the granuloma in expansion and dissemination of early tuberculous infection. Cell. 2009;136(1):37-49. Epub 2009/01/13. doi: 10.1016/j.cell.2008.11.014. PubMed PMID: 19135887; PubMed Central PMCID: PMCPMC3134310.
7. Houben RM, Dodd PJ. The Global Burden of Latent Tuberculosis Infection: A Re-estimation Using Mathematical Modelling. PLoS Med. 2016;13(10):e1002152. Epub 2016/10/26. doi: 10.1371/journal.pmed.1002152. PubMed PMID: 27780211; PubMed Central PMCID: PMCPMC5079585.
8. Azizi F, Hatami H, Janghorbani MJTEP. Epidemiology and control of common diseases in Iran. 2000:602-16.
9. Joshi R, Reingold AL, Menzies D, Pai MJPm. Tuberculosis among health-care workers in low- and middle-income countries: a systematic review. 2006;3(12):e494.
10. Kassim S, Zuber P, Wiktor S, Diomande F, Coulibaly I, Coulibaly D, et al. Tuberculin skin testing to assess the occupational risk of Mycobacterium tuberculosis infection among health care workers in Abidjan, Cote d'Ivoire. 2000;4(4):321-6.
11. Nosocomial TJIJT. Nosocomial tuberculosis in the era of drug resistant tuberculosis. 2009;56:59-61.
12. Yanai H, Limpakarnjanarat K, Uthaivoravit W, Mastro T, Mori T, Tappero JJTIJoT, et al. Risk of Mycobacterium tuberculosis infection and disease among health care workers, Chiang Rai, Thailand. 2003;7(1):36-45.
13. Field MJ. The occupational tuberculosis risk of health care workers. Tuberculosis in the Workplace: National Academies Press (US); 2001.
14. Jasmer RM, Nahid P, Hopewell PCJNEJoM. Latent tuberculosis infection. 2002;347(23):1860-6.
15. Christopher DJ, Daley P, Armstrong L, James P, Gupta R, Premkumar B, et al. Tuberculosis infection among young nursing trainees in South India. 2010;5(4):e10408.
16. Garber E, San Gabriel P LL, Saiman L. A survey of latent tuberculosis infection among laboratory healthcare workers in New York City. Infection Control & Hospital Epidemiology. 2003;24(11):801-6.
17. Jo K-W, Hong Y, Park JS, Bae I-G, Eom JS, Lee S-R, et al. Prevalence of latent tuberculosis infection among health care workers in South Korea: a multicenter study. 2013;75(1):18-24.
18. Brock I, Weldingh K, Lillebaek T, Follmann F, Andersen P. Comparison of tuberculin skin test and new specific blood test in tuberculosis contacts. Am J Respir Crit Care Med. 2004;170(1):65-9. Epub 2004/04/17. doi: 10.1164/rccm.200402-232OC. PubMed PMID: 15087297.
19. Kang YA, Lee HW, Yoon HI, Cho B, Han SK, Shim Y-S, et al. Discrepancy between the tuberculin skin test and the whole-blood interferon γ assay for the diagnosis of latent tuberculosis infection in an intermediate tuberculosis-burden country. 2005;293(22):2756-61.

20. Mori T, Sakatani M, Yamagishi F, Takashima T, Kawabe Y, Nagao K, et al. Specific detection of tuberculosis infection: an interferon-γ–based assay using new antigens. 2004;170(1):59-64.
21. Bennett JE, Dolin R, Blaser MJ. Mandell, Douglas, and Bennett's Principles and Practice of Infectious Diseases: 2-Volume Set: Elsevier Health Sciences; 2014.
22. Menzies DJCID. What does tuberculin reactivity after bacille Calmette-Guérin vaccination tell us? 2000;31(Supplement_3):S71-S4.
23. Menzies R, Vissandjee B. Effect of bacille Calmette-Guerin vaccination on tuberculin reactivity. Am Rev Respir Dis. 1992;145(3):621-5. Epub 1992/03/01. doi: 10.1164/ajrccm/145.3.621. PubMed PMID: 1546843.
24. Gazi MA, Islam MR, Kibria MG, Mahmud ZJEJoCM, Diseases I. General and advanced diagnostic tools to detect Mycobacterium tuberculosis and their drug susceptibility: a review. 2015;34(5):851-61.
25. Nasehi M, Hashemi-Shahraki A, Doosti-Irani A, Sharafi S, Mostafavi EJE, health. Prevalence of latent tuberculosis infection among tuberculosis laboratory workers in Iran. 2017;39.
26. Liberati A, Taricco MJRiP, Books RMPMF. How to do and report systematic reviews and meta-analysis. 2010:137-64.
27. Cook DJ, Mulrow CD, Haynes RB. Systematic reviews: synthesis of best evidence for clinical decisions. Ann Intern Med. 1997;126(5):376-80. Epub 1997/03/01. PubMed PMID: 9054282.
28. Moher D, Liberati A, Tetzlaff J, Altman DGJAoim. Preferred reporting items for systematic reviews and meta-analyses: the PRISMA statement. 2009;151(4):264-9.
29. Editors PLM. Best practice in systematic reviews: the importance of protocols and registration. PLoS Med. 2011;8(2):e1001009. Epub 2011/03/03. doi: 10.1371/journal.pmed.1001009. PubMed PMID: 21364968; PubMed Central PMCID: PMCPMC3042995.
30. Nasiri MJ, Pormohammad A, Goudarzi H, Mardani M, Zamani S, Migliori GB, et al. Latent tuberculosis infection in transplant candidates: a systematic review and meta-analysis on TST and IGRA. Infection. 2019;47(3):353-61. Epub 2019/02/26. doi: 10.1007/s15010-019-01285-7. PubMed PMID: 30805899.
31. JafariNezhad A, YektaKooshali MH. Lung cancer in idiopathic pulmonary fibrosis: A systematic review and meta-analysis. PLoS One. 2018;13(8):e0202360. Epub 2018/08/17. doi: 10.1371/journal.pone.0202360. PubMed PMID: 30114238; PubMed Central PMCID: PMCPMC6095562.
32. da Costa Santos CM, de Mattos Pimenta CA, Nobre MR. The PICO strategy for the research question construction and evidence search. Rev Lat Am Enfermagem. 2007;15(3):508-11. Epub 2007/07/27. PubMed PMID: 17653438; PubMed Central PMCID: PMC17653438.
33. Krajewski W, Zdrojowy R, Grzegolka J, Krajewski P, Wrobel M, Luczak M, et al. Does Mantoux Test Result Predicts BCG Immunotherapy Efficiency and Severe ToXxicity in Non-Muscle Invasive Bladder Cancer. Urology journal. 2018. Epub 2018/11/25. doi: 10.22037/uj.v0i0.4542. PubMed PMID: 30471076.
34. Sargin G, Senturk T, Ceylan E, Telli M, Cildag S, Dogan H. TST, QuantiFERON-TB Gold test and T-SPOT.TB test for detecting latent tuberculosis infection in patients with rheumatic disease prior to anti-TNF therapy. Tuberkuloz ve toraks. 2018;66(2):136-43. Epub 2018/09/25. doi: 10.5578/tt.66444. PubMed PMID: 30246657.
35. Wang W, Liu HM, Zhou J, Wang YG, Feng X, Tang H, et al. Skin test of tuberculin purified protein derivatives with a dissolving microneedle-array patch. Drug delivery and translational research. 2019. Epub 2019/03/21. doi: 10.1007/s13346-019-00629-y. PubMed PMID: 30891708.
36. Wigg AJ, Narayana SK, Anwar S, Ramachandran J, Muller K, Chen JW, et al. High rates of indeterminate interferon-gamma release assays for the diagnosis of latent tuberculosis infection in liver transplantation candidates. Transplant infectious disease : an official journal of the Transplantation Society. 2019:e13087. Epub 2019/03/31. doi: 10.1111/tid.13087. PubMed PMID: 30927483.

37. Barton E, Gao Y, Ball D, Fidler K, Klein N, Curtis N, et al. Calcineurin Inhibitors and Variation in the Performance of Interferon-gamma Release Assays Used to Detect Tuberculosis Infection. Annals of the American Thoracic Society. 2019;16(6):771-5. Epub 2019/02/28. doi: 10.1513/AnnalsATS.201811-784RL. PubMed PMID: 30811214.
38. Bennet R, Nejat S, Eriksson M. Effective Tuberculosis Contact Investigation Using Interferon-Gamma Release Assays. The Pediatric infectious disease journal. 2019;38(4):e76-e8. Epub 2019/03/19. doi: 10.1097/INF.0000000000002272. PubMed PMID: 30882747.
39. Igari H, Akutsu N, Ishikawa S, Aoyama H, Otsuki K, Hasegawa M, et al. Positivity rate of interferon-gamma release assays for estimating the prevalence of latent tuberculosis infection in renal transplant recipients in Japan. Journal of infection and chemotherapy : official journal of the Japan Society of Chemotherapy. 2019. Epub 2019/03/25. doi: 10.1016/j.jiac.2019.02.018. PubMed PMID: 30905632.
40. Poorolajal J, Cheraghi Z, Irani AD, Rezaeian S. Quality of Cohort Studies Reporting Post the Strengthening the Reporting of Observational Studies in Epidemiology (STROBE) Statement. Epidemiol Health. 2011;33:e2011005. Epub 2011/07/01. doi: 10.4178/epih/e2011005. PubMed PMID: 21716598; PubMed Central PMCID: PMCPMC3110877.
41. Ades AE, Lu G, Higgins JP. The interpretation of random-effects meta-analysis in decision models. Med Decis Making. 2005;25(6):646-54. Epub 2005/11/12. doi: 10.1177/0272989X05282643. PubMed PMID: 16282215.
42. Borenstein M, Hedges LV, Higgins JP, Rothstein HR. A basic introduction to fixed-effect and random-effects models for meta-analysis. Res Synth Methods. 2010;1(2):97-111. Epub 2010/04/01. doi: 10.1002/jrsm.12. PubMed PMID: 26061376.
43. Alian S, Dadashi A, Najafi N, Alikhani A, Davoudi A, Moosazadeh M, et al. Evaluation of Tuberculin Skin Test (TST) in Medical Students in Mazandaran University of Medical Sciences, Sari, Iran. Global Journal of Health Science. 2016;9(5):274.
44. Besharat M, Abbasi F. Tuberculin Skin Test among 1,424 Healthy Employees in Chaharmahal Province, Iran. Tanaffos. 2011;1(10):37-9.
45. Davoodi L, Babamahmoodi F, Mirabi A, Mohammad Hosseini E. Evaluation of Tuberculin Skin Test Seroconversion Among the Staff in Qaemshahr Razi Hospital, 2015-2017. Journal of Mazandaran University of Medical Sciences. 2018;28(164):158-63.
46. Ghafouri M, Seyed Sharifi S. Prevalence of latent tuberculosis infections in Health care workers (HCW) in Mashhad Hospital. Journal of North Khorasan University of Medical Sciences. 2015;6(4):829-40.
47. Golchin M, Rostami M. Tuberculin test in nursing and human-sciences students. Journal of Research in Medical Sciences. 2005;10(3):172-6.
48. Hashemi SH, Mamani M, Alizadeh N, Nazari M, Sedighi I. Prevalence of tuberculosis infection among health-care workers in Hamadan, west of Iran. Avicenna Journal of Clinical Microbiology and Infection. 2014;1(1):e19214.
49. Kariminia A, Sharifnia Z, Aghakhani A, Banifazl M, Eslamifar A, Hazrati M, et al. Comparison of QuantiFERON TB-G-test to TST for detecting latent tuberculosis infection in a high-incidence area containing BCG-vaccinated population. Journal of evaluation in clinical practice. 2009;15(1):148-51. Epub 2009/02/26. doi: 10.1111/j.1365-2753.2008.00970.x. PubMed PMID: 19239595.
50. Mostafavi E, Nasehi M, Hashemi Shahraki A, Esmaeili S, Ghaderi E, Sharafi S, et al. Comparison of the tuberculin skin test and the QuantiFERON-TB Gold test in detecting latent tuberculosis in health care workers in Iran. Epidemiol Health. 2016;38:e2016032. Epub 2016/07/28. doi: 10.4178/epih.e2016032. PubMed PMID: 27457062; PubMed Central PMCID: PMCPMC5037355.
51. Nasehi M, Hashemi-Shahraki A, Doosti-Irani A, Sharafi S, Mostafavi E. Prevalence of latent tuberculosis infection among tuberculosis laboratory workers in Iran. Epidemiol Health. 2017;39:e2017002. Epub 2017/01/18. doi: 10.4178/epih.e2017002. PubMed PMID: 28092930; PubMed Central PMCID: PMCPMC5343107.

52. Nazer M, Shahivand M, Zare S. The prevalence of latent tuberculosis (TB) infection in healthcare staff of Khorramabad Ashayer hospital in 2015. Yafte. 2015;17(2):23-31.
53. Nikokar I, Dadgran A, Mafozei L. A comparison of two-step tuberculin skin test between health-care workers and nonhospital employees. Iranian Journal of Medical Sciences. 2015;35(3):201-4.
54. Rahbar M, Karamyar M, Hajia M. Prevalence and determinant of tuberculin skin test among health care workers of Imam Khomeini Hospital of Uremia, Iran. Shiraz E-Medical Journal. 2007;8(4):162-7.
55. Salehi M, Mood BS, Metanat M. Positive Tuberculin Skin Test Among Health Care Workers: Prevalence and Risk Factors in Teaching Hospitals of a Highly Endemic Region for Tuberculosis, Zahedan, Iran. International Journal of Infection. 2016;3(3).
56. Salmanzadeh S, Abbasissifar H, Alavi SM. Comparison study of QuantiFERON test with tuberculin skin testing to diagnose latent tuberculosis infection among nurses working in teaching hospitals of Ahvaz, Iran. Caspian journal of internal medicine. 2016;7(2):82-7. Epub 2016/07/08. PubMed PMID: 27386058; PubMed Central PMCID: PMCPMC4913709.
57. Sayad B, Zarpeyma A, Janbakhsh A. Tuberculin Skin Test Results in Health Care Workers of Imam Khomeini Hospital (Kermanshah 2004). Journal of Kermamnshah University of Medical Sciences(Behbood). 2006;10(3):258-67.
58. Sharafkhani R, Ahmadi N, Salarilak S, Rahimirad M, Khashabi J. prevalence of tuberculosis infection in health and office workers at Urmia University of Medical Sciences. Journal of Urmia University of Medical Sciences. 2011;22(2):119-22.
59. Taheri M, Bazrafkan H, Habibagahi M. Determining the Latent Tuberculosis Infection by IFN - gamma Elispot Assay in Healthcare Workers From University Hospitals of Shiraz, South West of Iran. Iranian Red Crescent medical journal. 2013;15(6):477-82. Epub 2013/12/19. doi: 10.5812/ircmj.3635. PubMed PMID: 24349745; PubMed Central PMCID: PMCPMC3840834.
60. Talebi-Taher M, Javad-Moosavi SA, Entezari AH, Shekarabi M, Parhizkar B. Comparing the performance of QuantiFERON-TB Gold and Mantoux test in detecting latent tuberculosis infection among Iranian health care workers. Int J Occup Med Environ Health. 2011;24(4):359-66. Epub 2011/11/17. doi: 10.2478/s13382-011-0046-7. PubMed PMID: 22086450.
61. Tavanaee Sani A, Hajian S, Maryam S. Evaluation of PPD test in Medical Student of Mashhad University Medical Sciences in 2011-2013. Medical Journal of Mashhad University of Medical Sciences. 2015;58(8):441-5.
62. Vaziri S, Khazaei S, Neishaboori S, Kanani M, Madani S. The degree of agreement of quantiferon TB gold test and tuberculin skin test in nurses. Journal of Gorgan University of Medical Sciences. 2011;13(1):37-43.
63. Agaya J, Nnadi CD, Odhiambo J, Obonyo C, Obiero V, Lipke V, et al. Tuberculosis and latent tuberculosis infection among healthcare workers in K isumu, K enya. 2015;20(12):1797-804.
64. Corbett EL, Marston B, Churchyard GJ, De Cock KM. Tuberculosis in sub-Saharan Africa: opportunities, challenges, and change in the era of antiretroviral treatment. Lancet. 2006;367(9514):926-37. Epub 2006/03/21. doi: 10.1016/S0140-6736(06)68383-9. PubMed PMID: 16546541.
65. Drobniewski F, Cooke M, Jordan J, Casali N, Mugwagwa T, Broda A, et al. Systematic review, meta-analysis and economic modelling of molecular diagnostic tests for antibiotic resistance in tuberculosis. Health Technol Assess. 2015;19(34):1-188, vii-viii. Epub 2015/05/09. doi: 10.3310/hta19340. PubMed PMID: 25952553; PubMed Central PMCID: PMCPMC4781420.
66. Franco C, Zanetta DJTIJoT, Disease L. Assessing occupational exposure as risk for tuberculous infection at a teaching hospital in Sao Paulo, Brazil. 2006;10(4):384-9.
67. Powell K, Han D, Hung N, Vu T, Sy D, Trinh T, et al. Prevalence and risk factors for tuberculosis infection among personnel in two hospitals in Viet Nam. 2011;15(12):1643-9.

68. Rutanga C, Lowrance DW, Oeltmann JE, Mutembayire G, Willis M, Uwizeye CB, et al. Latent tuberculosis infection and associated factors among Health Care Workers in Kigali, Rwanda. 2015;10(4):e0124485.
69. Zhu S, Xia L, Yu S, Chen S, Zhang J. The burden and challenges of tuberculosis in China: findings from the Global Burden of Disease Study 2015. Sci Rep. 2017;7(1):14601. Epub 2017/11/04. doi: 10.1038/s41598-017-15024-1. PubMed PMID: 29097809; PubMed Central PMCID: PMCPMC5668247.
70. van Rie A, Beyers N, Gie RP, Kunneke M, Zietsman L, Donald PR. Childhood tuberculosis in an urban population in South Africa: burden and risk factor. Arch Dis Child. 1999;80(5):433-7. Epub 1999/04/20. doi: 10.1136/adc.80.5.433. PubMed PMID: 10208948; PubMed Central PMCID: PMCPMC1717911.
71. Durando P, Sotgiu G, Spigno F, Piccinini M, Mazzarello G, Viscoli C, et al. Latent tuberculosis infection and associated risk factors among undergraduate healthcare students in Italy: a cross-sectional study. 2013;13(1):443.
72. Storla DG, Yimer S, Bjune GA. A systematic review of delay in the diagnosis and treatment of tuberculosis. BMC Public Health. 2008;8(1):15. Epub 2008/01/16. doi: 10.1186/1471-2458-8-15. PubMed PMID: 18194573; PubMed Central PMCID: PMCPMC2265684.
73. Prasad R, Singh A, Balasubramanian V, Gupta N. Extensively drug-resistant tuberculosis in India: Current evidence on diagnosis & management. Indian J Med Res. 2017;145(3):271-93. Epub 2017/07/28. doi: 10.4103/ijmr.IJMR_177_16. PubMed PMID: 28749390; PubMed Central PMCID: PMCPMC5555056.
74. Nasreen S, Shokoohi M, Malvankar-Mehta MSJPo. Prevalence of latent tuberculosis among health care workers in high burden countries: a systematic review and meta-analysis. 2016;11(10):e0164034.
75. Apriani L, McAllister S, Sharples K, Alisjahbana B, Ruslami R, Hill PC, et al. Latent tuberculosis infection in health care workers in low and middle-income countries: an updated systematic review. 2019:1801789.
76. Karadakhy K, Othman N, Ibrahimm F, Saeed AA, Amin AAH. Tuberculosis in Sulaimaniyah, Iraqi Kurdistan: A Detailed Analysis of Cases Registered in Treatment Centers. Tanaffos. 2016;15(4):197-204. Epub 2016/01/01. PubMed PMID: 28469675; PubMed Central PMCID: PMCPMC5410115.
77. Hassan DN, Hanna AJ. Tuberculosis and sudden death in Baghdad. Am J Forensic Med Pathol. 1984;5(2):169-74. Epub 1984/06/01. PubMed PMID: 6731410.
78. Sargazi A, Sepehri Z, Sagazi A, Jim PN, Kiani ZJAr, control i. Eastern Mediterranean region tuberculosis economic burden in 2014. 2015;4(1):P102.
79. Sahebi L, Ansarin K, Maryam S, Monfaredan A, Sabbgh Jadid H. The factors associated with tuberculosis recurrence in the northwest and west of iran. Malays J Med Sci. 2014;21(6):27-35. Epub 2015/04/22. PubMed PMID: 25897280; PubMed Central PMCID: PMCPMC4391452.
80. Jimma W, Ghazisaeedi M, Shahmoradi L, Abdurahman AA, Kalhori SRN, Nasehi M, et al. Prevalence of and risk factors for multidrug-resistant tuberculosis in Iran and its neighboring countries: systematic review and meta-analysis. Rev Soc Bras Med Trop. 2017;50(3):287-95. Epub 2017/07/13. doi: 10.1590/0037-8682-0002-2017. PubMed PMID: 28700044.
81. Rezai MS, Abedi S, Afshari M, Moosazadeh MJOph, perspectives r. Estimating tuberculin skin test reactions among children and teenagers who received the bacillus Calmette-Guerin vaccination at birth: A meta-analysis. 2017;8(1):3.
82. Goscé L, Bitencourt J, Gupta R, Arruda S, Rodrigues L, Abubakar IJIJoID. BCG vaccination following latent TB treatment: Possible implications for different settings. 2019;80:S17-S9.
83. Al-Orainey IOJAotm. Diagnosis of latent tuberculosis: Can we do better? 2009;4(1):5.

11. Supporting information

S1 File. PRISMA Checklist

S2 File. The review protocol which has been registered in PROSPERO International Prospective Register of Systematic Reviews

S3 File. Newcastle-Ottawa scale checklist

S1 Table. Data characteristics (Full details) (MS Excel)

S1 Fig. The prevalence subgroup analysis (Forest plot - Random effect model) of TST/PPD induration diameter (48 hrs.) in HCWs with LTBI.

S2 Fig. The prevalence subgroup analysis (Forest plot - Random effect model) of TST/PPD induration diameter (48 hrs.) in HCWs with LTBI base on geographical region.

S3 Fig. Cumulative meta-analysis for overall prevalence of LTBI in HCWs.

S4 Fig. The prevalence subgroup analysis to employment duration (Forest plot - Random effect model) base on PPD (A), and QFT (B) in HCWs with LTBI.

S5 Fig. The prevalence subgroup analysis of gender (Forest plot - Random effect model) base on PPD (A), and QFT (B) in HCWs with LTBI.

S6 Fig. The prevalence subgroup analysis of age (Forest plot - Random effect model) base on PPD (A), and QFT (B) in HCWs with LTBI.

S7 Fig. The prevalence subgroup analysis of history TB contact (Forest plot - Random effect model) base on PPD (A), and QFT (B) in HCWs with LTBI.

S8 Fig. The prevalence subgroup analysis of TB clinical symptoms (Forest plot - Random effect model) base on PPD (A), and QFT (B) in HCWs with LTBI.

S9 Fig. The prevalence subgroup analysis of BCG (Forest plot - Random effect model) base on PPD (A), and QFT (B) in HCWs with LTBI.

S10 Fig. Publication bias of studies included due to the aim of prevalence of HCWs with LTBI.

12. Appendix: PubMed search strategy:

((Latent Tuberculosis) AND Iran AND Prevalence AND ((Health Personnel) OR (Healthcare Worker) OR (Health Care Provider))

Figure 1

Click here to access/download;Figure;Fig 1.JPG

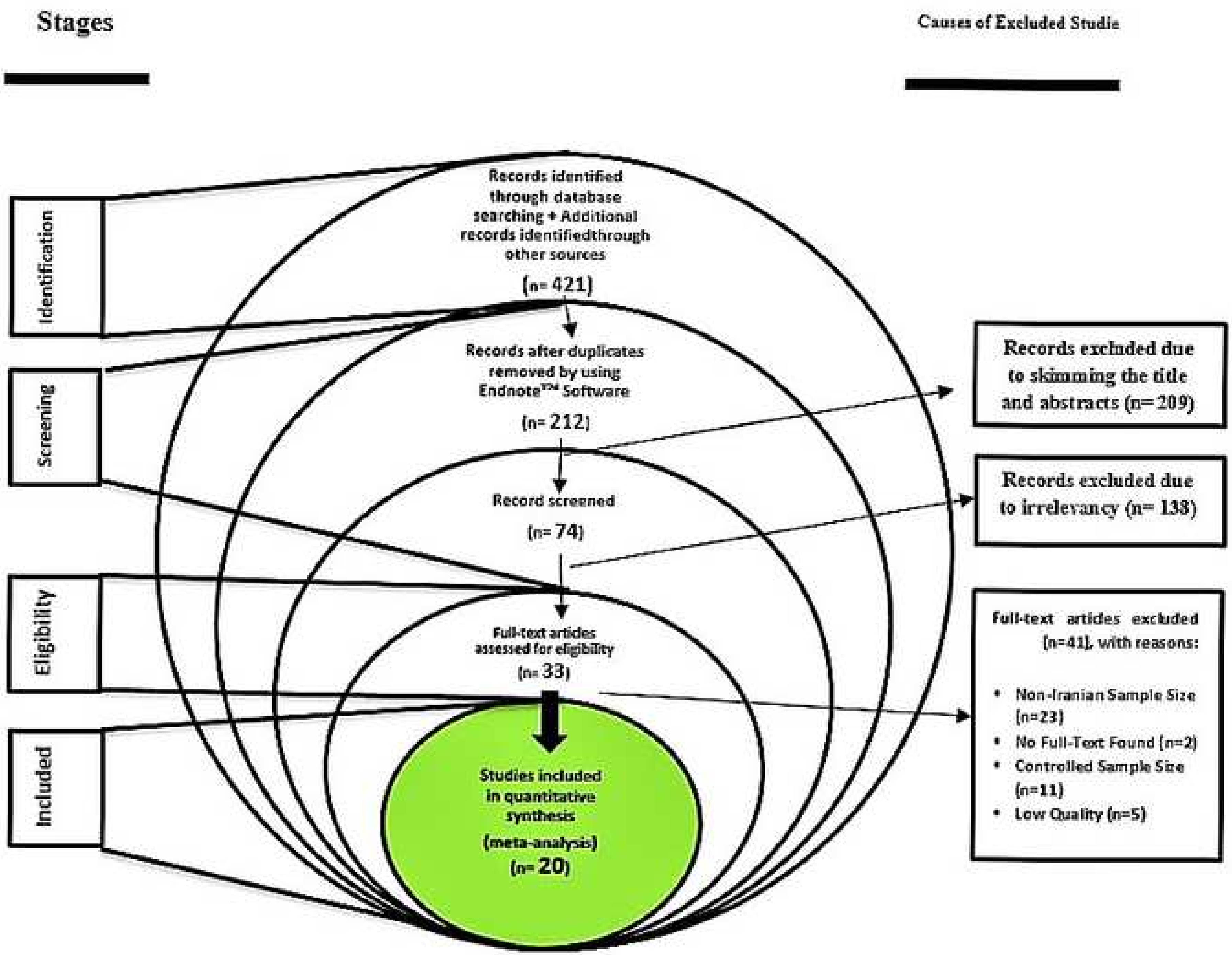

Figure 2 Click here to access/download;Figure;Fig 2.tif

Study name	Statistics for each study					Weight (Random)	
	Event rate	Lower limit	Upper limit	Z-Value	p-Value	Total	Relative weight
Taheri et al. (2013)	0.0787	0.0380	0.1559	-6.2494	0.0000	7 / 89	5.94
Davoodi et al., (2018)	0.1140	0.0674	0.1865	-6.9578	0.0000	13 / 114	6.36
Tavanaee Sani et al.(2015)	0.0650	0.0381	0.1087	-9.2953	0.0000	13 / 200	6.39
Vaziri et al., (2011)	0.3478	0.2453	0.4667	-2.4870	0.0129	24 / 69	6.51
Salmanzadeh et al.,(2016)	0.3563	0.2631	0.4619	-2.6416	0.0083	31 / 87	6.61
Alian et al., (2016)	0.1077	0.0772	0.1484	-11.2960	0.0000	32 / 297	6.72
Mostafavi et al., (2016)	0.1639	0.1226	0.2158	-9.4218	0.0000	40 / 244	6.76
nazer et al. (2015)	0.1794	0.1344	0.2353	-8.7120	0.0000	40 / 223	6.75
Ghafouri et al.,(2015)	0.1492	0.1199	0.1841	-13.5333	0.0000	71 / 476	6.86
Kariminia et al. (2009)	0.4830	0.4100	0.5566	-0.4522	0.6511	85 / 176	6.81
Talebi-Taher et al. (2011)	0.5250	0.4558	0.5933	0.7068	0.4797	105 / 200	6.83
SharafKhani et al. (2011)	0.3579	0.3055	0.4138	-4.8464	0.0000	107 / 299	6.87
Nikokar et al. (2010)	0.6108	0.5387	0.6784	2.9890	0.0028	113 / 185	6.81
Sayad et al. (2006)	0.5063	0.4515	0.5609	0.2243	0.8225	161 / 318	6.89
Salehi et al. (2016)	0.5671	0.5129	0.6197	2.4221	0.0154	186 / 328	6.89
	0.2713	0.1864	0.3770	-3.9872	0.0001		

Event rate and 95% CI

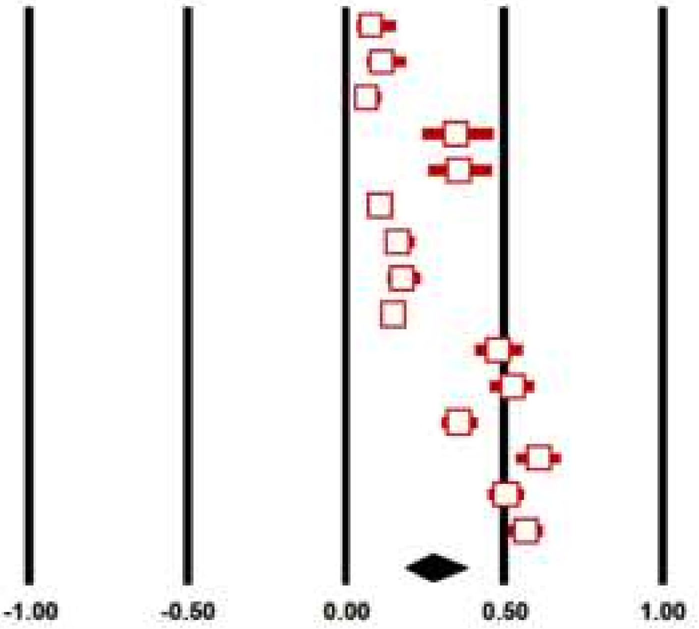

Meta Analysis

Figure 3

Click here to access/download;Figure;Fig 3.tif

Study name	Statistics for each study						Weight (Random)
	Event rate	Lower limit	Upper limit	Z-Value	p-Value	Total	Relative weight
Kariminia et al. (2009)	0.0795	0.0477	0.1298	-8.7897	0.0000	14 / 176	19.29
Talebi-Taher et al. (2011)	0.0850	0.0535	0.1325	-9.3720	0.0000	17 / 200	19.76
Vaziri et al., (2011)	0.3043	0.2076	0.4222	-3.1597	0.0016	21 / 69	19.61
Salmanzadeh et al.,(2016)	0.3103	0.2222	0.4148	-3.4457	0.0006	27 / 87	20.16
Mostafavi et al., (2016)	0.1762	0.1334	0.2292	-9.1781	0.0000	43 / 244	21.18
	0.1692	0.0970	0.2784	-4.8789	0.0000		

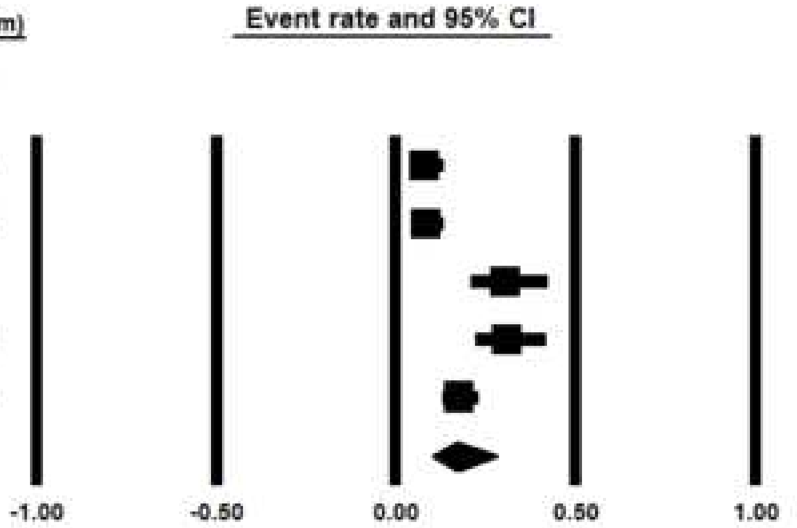

Meta Analysis

Figure 4

Click here to access/download;Figure;Fig 4.tif

Study name	Statistics for each study					Weight (Random)	
	Event rate	Lower limit	Upper limit	Z-Value	p-Value	Total	Relative weight
Davoodi et al., (2018)	0.4158	0.3240	0.5140	-1.6834	0.0923	42 / 101	21.47
Alian et al., (2016)	0.0755	0.0492	0.1141	-10.7739	0.0000	20 / 265	21.27
Seyad et al. (2006)	0.0233	0.0058	0.0682	-5.2240	0.0000	2 / 86	16.00
Nikokar et al. (2010)	0.1846	0.1079	0.2976	-4.6463	0.0000	12 / 65	20.56
Salehi et al. (2016)	0.0923	0.0532	0.1565	-7.5439	0.0000	12 / 130	20.71
	0.1211	0.0453	0.2857	-3.6454	0.0003		

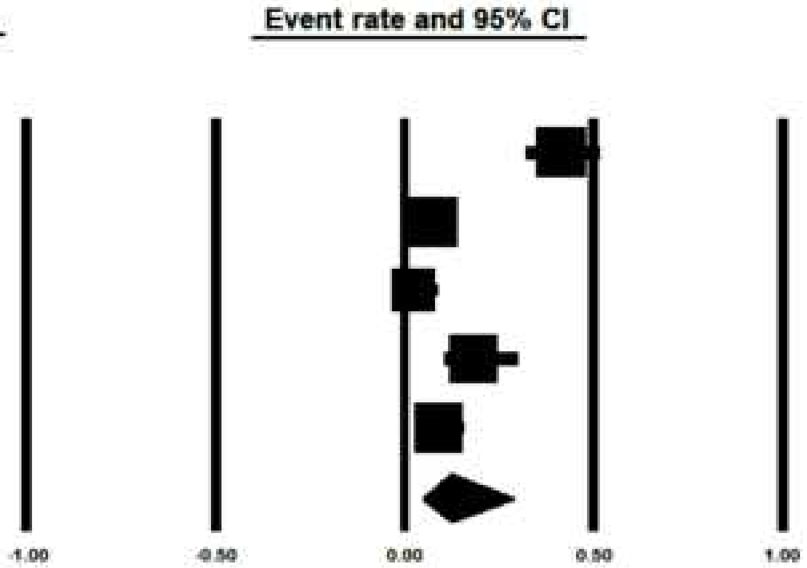

Meta Analysis

Figure 5 Click here to access/download;Figure;Fig 5.tif

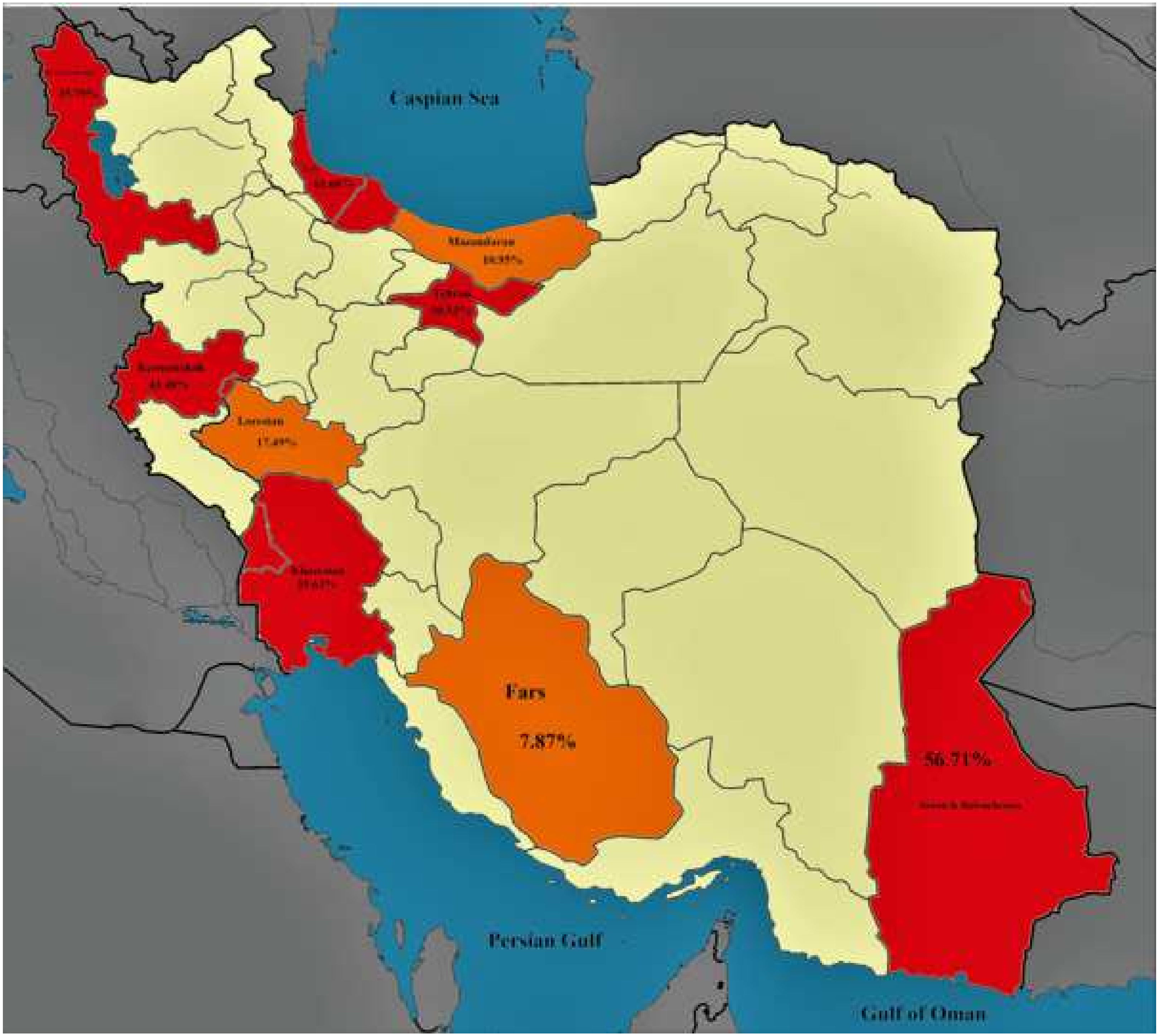

Figure 6

Click here to access/download;Figure;Fig 6.tif

Study name	Statistics with study removed				
	Point	Lower limit	Upper limit	Z-Value	p-Value
Taheri et al. (2013)	0.2902	0.1998	0.4009	-3.5579	0.0004
Davoodi et al., (2018)	0.2859	0.1961	0.3965	-3.6221	0.0003
Tavanaee Sani et al. (2015)	0.2949	0.2055	0.4034	-3.5555	0.0004
Vaziri et al., (2011)	0.2662	0.1788	0.3767	-3.8926	0.0001
Salmanzadeh et al.,(2016	0.2656	0.1780	0.3765	-3.8870	0.0001
Alian et al., (2016)	0.2881	0.2003	0.3955	-3.6905	0.0002
Mostafavi et al., (2016)	0.2806	0.1913	0.3914	-3.6897	0.0002
nazer et al. (2015)	0.2790	0.1894	0.3904	-3.6921	0.0002
Ghafouri et al.,(2015)	0.2829	0.1955	0.3902	-3.7675	0.0002
Kariminia et al. (2009)	0.2579	0.1716	0.3684	-4.0008	0.0001
Talebi-Taher et al. (2011)	0.2556	0.1705	0.3645	-4.0828	0.0000
SharafKhani et al. (2011)	0.2648	0.1739	0.3813	-3.7264	0.0002
Nikokar et al. (2010)	0.2511	0.1696	0.3551	-4.3192	0.0000
Sayad et al. (2006)	0.2564	0.1700	0.3674	-4.0047	0.0001
Salehi et al. (2016)	0.2533	0.1705	0.3589	-4.2299	0.0000
	0.2713	0.1864	0.3770	-3.9872	0.0001

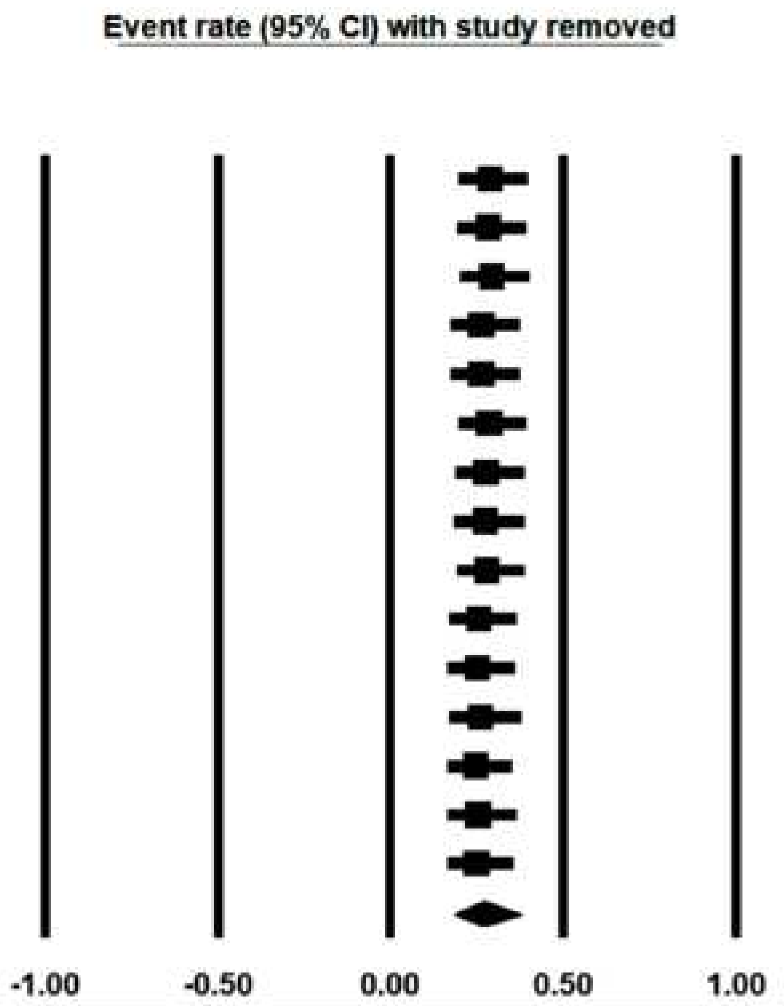

Meta Analysis

Figure 7

Click here to access/download;Figure;Fig 7.tif

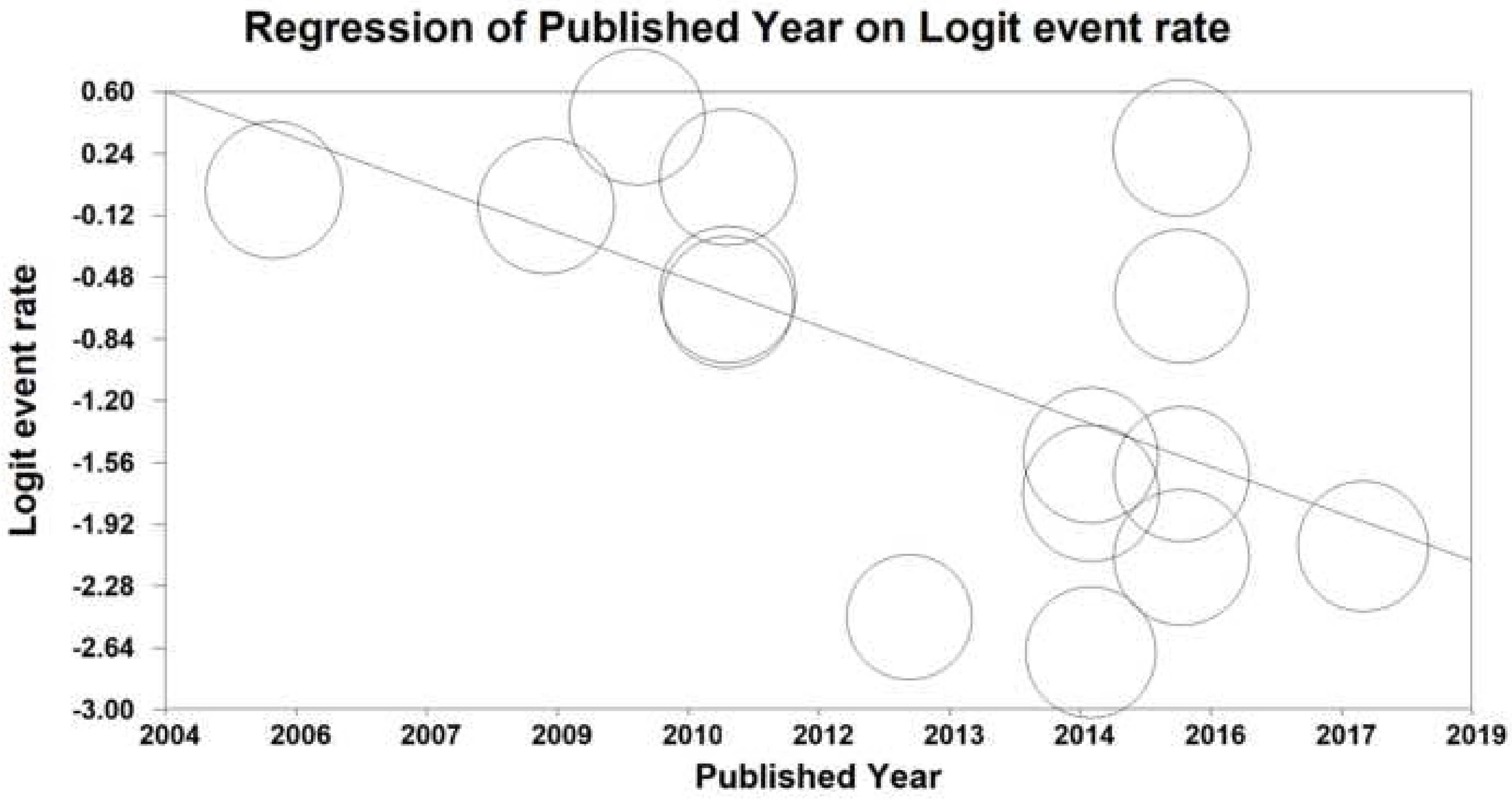

(Mixed effects regression (Method of moments); Slope = -0.1898(SE = 0.068, (95% CI: -0.323– -0.056)), Intercept = 381.14(SE = 137.43, (95% CI: 111.78–650.5)), P = 0.10653)

Figure 8

Click here to access/download;Figure;Fig 8.tif

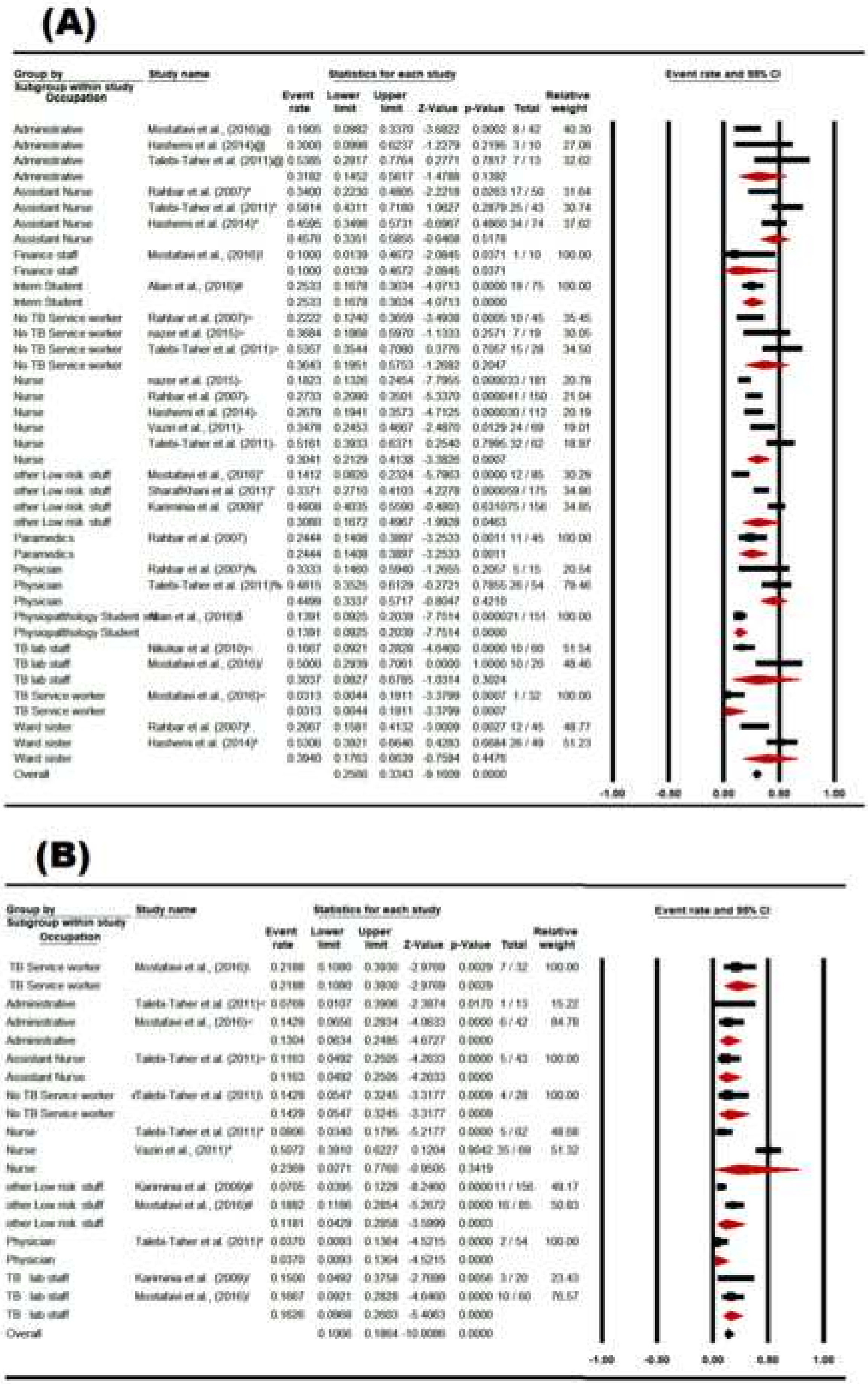

Figure 9

Click here to access/download;Figure;Fig 9.tif

(A)

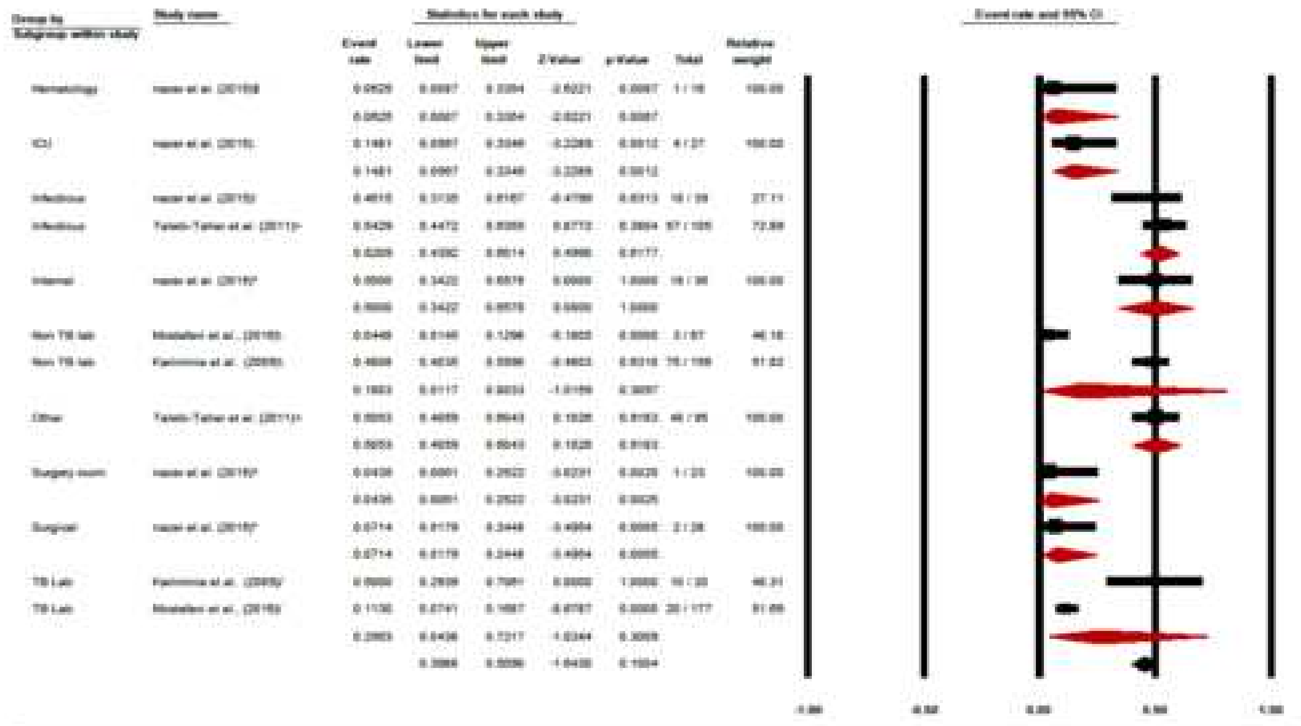

(B)

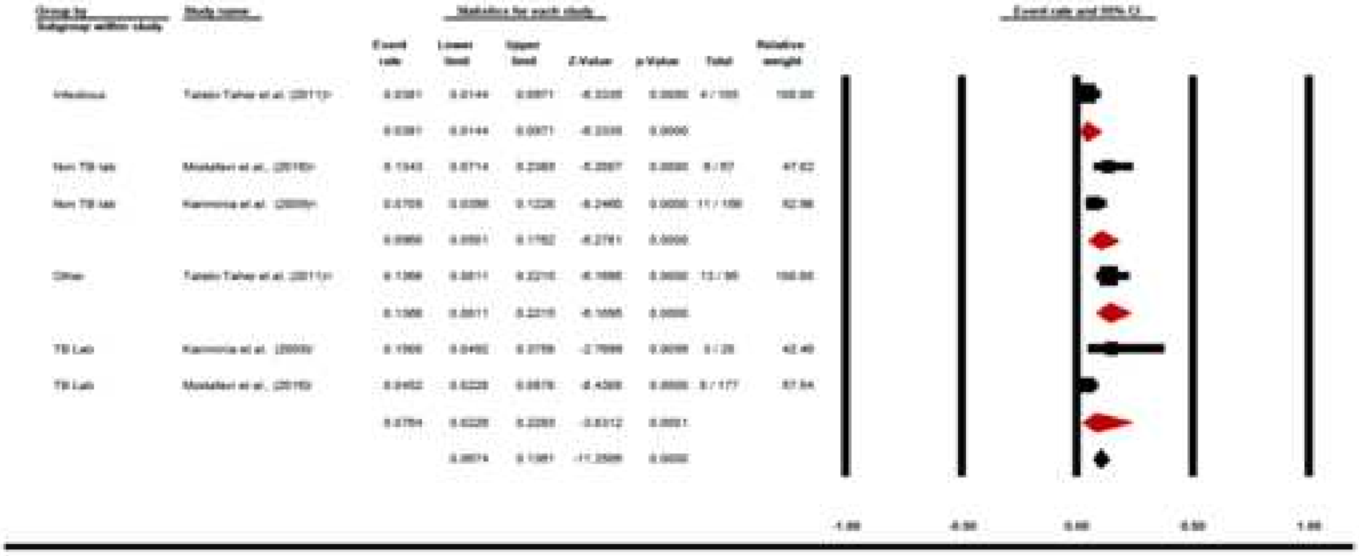

Supporting Information 1

Click here to access/download
Supporting Information
S1 Fig.tif

S1 File

Click here to access/download
Supporting Information
renamed_66a0a.DOC

S1 Table

Click here to access/download
Supporting Information
S1 Table.xlsx

S2 Fig

Click here to access/download
Supporting Information
S2 Fig.tif

S2 File

Click here to access/download
Supporting Information
renamed_b43ee.pdf

S3 Fig

Click here to access/download
Supporting Information
S3 Fig.tif

S3 File

Click here to access/download
Supporting Information
S3 File.pdf

S4 Fig

Click here to access/download
Supporting Information
S4 Fig.tif

S5 Fig

Click here to access/download
Supporting Information
S5 Fig.tif

S6 Fig

Click here to access/download
Supporting Information
S6 Fig.tif

S7 Fig

Click here to access/download
Supporting Information
S7 Fig.tif

S8 Fig

Click here to access/download
Supporting Information
S8 Fig.tif

S9 Fig

Click here to access/download
Supporting Information
S9 Fig.tif

S10 Fig

Click here to access/download
Supporting Information
S10 Fig.tif
