# Supplementary material for: Is latent tuberculosis infection challenging in Iranian health care workers? A systematic review and meta-analysis
Source: PLoS One. 2019 Oct 3;14(10):e0223335. doi: 10.1371/journal.pone.0223335 (PMC6776393; doi:10.1371/journal.pone.0223335)
Supplement: Attachment — Submitted filename: Response to Reviewers.docx [file pone.0223335.s017.docx]

September 3, 2019

*Dear Prof. Seyed Ehtesham Hasanian*,

We would like to thank you for the letter dated August 30, 2019 at 21:20, and the opportunity to resubmit a revised copy of this manuscript. We would also like to take this opportunity to express our thanks to the reviewers for the positive feedback and helpful comments for correction and modification. We believe have resulted in an improved revised manuscript, which you will find uploaded alongside this document. The manuscript has been revised to address the reviewer comments, which are appended alongside our responses to this letter.

We revised and provided this manuscript based on your journal author guidelines. *Dr. A.J (Blinded)* (as a Lead Author) affirms that this manuscript is an honest, accurate, and transparent account of the study being reported; that no important aspects of the study have been omitted; and that any discrepancies from the study as planned have been explained. All authors have read the revised manuscript and they are in agreement with its contents. Also, all authors declare no conflict of interests.

I want you to do us a favor and consider kindly our revised manuscript for publication in your very great academic journal and all authors are ready to edit the manuscript according to reviewers and your comments’.

Best Regards,

**Is latent tuberculosis infection challenging in Iranian health care workers? A systematic review and meta-analysis**

Manuscript ID: PONE-D-19-18112

Editor Recommendations:

1. Please ensure that your manuscript meets PLOS ONE's style requirements, including those for file naming. The PLOS ONE style templates can be found at

**Comment:** It was checked.

2. We suggest you thoroughly copyedit your manuscript for language usage, spelling, and grammar. If you do not know anyone who can help you do this, you may wish to consider employing a professional scientific editing service.

**Comment:** The English language, spelling, and grammar of manuscript was rechecked by Dr. Farahnaz Movahedzadeh as a Research Assistance Professor of UIC.

3. We note that Figure 5 in your submission contain a map image which may be copyrighted.

**Comment:** The map image which we used in this manuscript depicted by authors and we didn’t copy from other sources.

**Reviewer #1 recommendations:**

Health care workers (HCWs) are at ‎increased risk of latent tuberculosis infection ‎‎(LTBI). This study is aimed at conducting a ‎systematic review and meta-analysis of the ‎prevalence and incidence of LTBI in Iranian ‎HCWs. English throughout the manuscript is ‎flawed and makes it a difficult read. However, ‎authors have done meticulous meta-analysis. ‎Portions which require correction have been ‎highlighted in yellow in the attached pdf. The whole manuscript needs to be revamped ‎majorly and revised then based on scientific ‎criteria.

**Comment:** Background section line 28 ‎‎“changed” does not make any sense.

**Response**: The background section line 28 was improved to “The high chances of getting latent tuberculosis infection (LTBI) among health care workers (HCWs) will an enormous problem in low and upper-middle-income countries.”

**Comment:** Was there any period from when studies ‎were collected?

‎**Response**: Absolutely yes. The time interval of this study was without any time limitation till January 01, 2019.

**Comment:** Line 64, provide the range of prevalence in ‎other reports

**‎Response:** It was improved to “There are several reports of TB outbreaks in Iran. According to the Iranian’s ministry of health, the incidence and the prevalence of TB are high in Sistan and Baluchestan, Khorasan, Mazandaran, Guilan, West and East Azerbaijan, Ardabil, Kurdistan, Khuzestan and southern coasts. Conversely, the incidence and the prevalence of TB are low in the central parts of Iran. The highest incidence and prevalence of TB belong to Golestan and Sistan-Baluchistan.”

**Comment:** Line 69, change “transfer” to “spread”

**‎Response:** It was done.

**Comment:** Mention the number of low, medium and ‎high quality studies

‎**Response**: The quality scores of studies were mentioned in Table S1.

**Comment:** Line 190, “it was considered significant at ‎p<0.05

‎**Response:** It was done.

**Comment:** Line 234-239 not clear

**‎Response:** It was improved to ” In the PPD test, the prevalence of LTBI in Iranian’s HCWs with more than 10 years old work-experience was evaluated 51%. The prevalence of LTBI in Iranian’s HCWs with less than 10 years old work-experience was estimated at 29.30%. In the QFT test, the prevalence of LTBI in Iranian’s HCWs with more than 20 years old work-experience was calculated 20.49% [CI95%: 11-34.97], which showed a significant relationship between the duration of employment (P <0.0001) (S4 Fig)”.

**Comment:** Line 276, add p value

**‎Response:** It was done.

**Comment:** Line 308, rephrase

‎**Response:** It was done.

**Comment:** Did the prevalence estimates correlate to ‎WHO incidence rates?

‎**Response:** In this study, we just estimated the prevalence of LTBI among Iranian HCWs, not the incidence.

**Comment:** Legends for all figures need to be ‎improved with the aim of giving correct aim of ‎the experiment and its analysis

‎**Response:** It was done.

**Comment:** Line 263 says “there was no significant ‎relationship” and then gives p<0.0001 which is ‎contradictory

‎**Response:** It was improved to **“**The highest prevalence of LTBI in Iranian HCWs aged 30 years old was estimated 22.52% [CI95 %: 3.7-68.34] in the QFT. In both PPD test and QFT, it was evaluated that there was significant relationship between the prevalence of LTBI in HCWs, and age of HCWs (P<0.0001) (S6 Fig).”

**Comment:** What kind of studies have been included ‎cohort/cross sectional/ case studies?

‎**Response:** The cross sectional and cohort studies have been included.

**Comment:** In methodology section, which software ‎package was used for sensitivity test?

‎**Response:** Comprehensive Meta-Analysis (Ver 2 , Englewood, NJ 07631, USA)‏.

**Comment:** It is suggested that a more critical account ‎be provided in the discussion section taking ‎into account salient findings, problems and ‎gaps

‎**Response:** In light of the evidence, we preferred to focus more on the prevalence of LTBI among HCWs in the eastern provinces of Iran, specially provinces which neighbor of Iraq.

**Comment:** Check the entire MS for repetitions

**‎Response:** It was done.

**Reviewer #2 recommendations:**

The meta-analysis performed in ‎the manuscript shows the highest prevalence ‎of Latent TB infection in health care workers in ‎the north and the west of Iran due to ‎neighboring countries like Azerbaijan and Iraq, ‎respectively. Hence, there is a need to create ‎awareness in Iranian HCWs about isolation and ‎personal protection. However, the study handled a very serious issue ‎but following points need to be addressed

‎ **Comment:** Please follow the proper PRISMA template ‎and not a Venn Diagram

**‎Response:** It was done.

‎ **Comment:** What is the basis of sensitivity analysis is ‎not clear. What is the rationale for removing ‎that one study?

‎‎**Response:** According to the Cochrane hand book, by creating a given set of variables, an analyst can determine how changes in one variable affect the outcome. In other words, Sensitivity analysis is a method for predicting the outcome of a decision if a situation turns out to be different compared to the key predictions. It helps in assessing the riskiness of a strategy. Helps in identifying how dependent the output is on a particular input value.

<https://handbook-5-1.cochrane.org/chapter_9/9_7_sensitivity_analyses.htm>

It used a lot in other same meta-analysis which is published.

<https://www.sciencedirect.com/science/article/abs/pii/S1871402119303960>

<https://journals.plos.org/plosone/article?id=10.1371/journal.pone.0214738>

<https://journals.plos.org/plosone/article?id=10.1371/journal.pone.0164769>

<http://ijn.mums.ac.ir/article_13419.html>

<https://www.ncbi.nlm.nih.gov/pubmed/30573555>

**Comment:** The S2 figure is more important than the ‎Figure 5 with map. Maybe the map can go in ‎supplementary

‎‎**Response:** We appreciate your valuable comment, but we believe that the map could be more clearer and it could convey the author's intent to the readers.

**Comment:** As they say national databases are not ‎sensitive to Boolean operators, do the authors ‎think this affects the search? Does this mean it ‎is not an exhaustive search on this topic?

‎**Response:** Thank you for your attention. In national databases, the SID is ‎ just sensitive to Boolean operators. So, we searched other Persian national databases (Barakat knowledge network system; Irandoc, Magiran, and Iranian national library) through manual ways.

**Comment:** Doing a sensitivity analysis based on the ‎quality of study will also add value.

‎‎**Response:** The sub group analysis of the ‎quality of studies was showed in (S4 Fig).

**Comment:** The search strategy provided by the authors ‎is very broad. They do not include the ‎tests, they do not include different terms for ‎HCWs, eg: T-SPOT, community workers, TST ‎etc. I do not feel this is a complete and ‎detailed search.

**Response:** Thanks to your valuable comment. To including of different terms of LTBI in Iranian ‘s HCWs. we searched more synonymous of keywords in the national databases.

**Comment:** Was data extracted for both one-step and ‎two-step TST? If so, doing that analysis is also ‎important. The authors say they did it but is not ‎available. ‎

**‎ Response:** As shown in Table S1, only two studies extracted one-step and two-step TST data that could not be analyzed.
